# Correction to “Comparing Scientific Machine Learning With Population Pharmacokinetic and Classical Machine Learning Approaches for Prediction of Drug Concentrations”

**DOI:** 10.1002/psp4.70024

**Published:** 2025-03-26

Valderrama, D. , Teplytska, O. , Koltermann, L.M. , Trunz, E. , Schmulenson, E. , Fritsch, A. , Jaehde, U. and Fröhlich, H. (2025), Comparing Scientific Machine Learning With Population Pharmacokinetic and Classical Machine Learning Approaches for Prediction of Drug Concentrations. CPT Pharmacometrics Syst Pharmacol. 10.1002/psp4.13313 PMC1200127539921335

In the published version of the above article, we noticed an inaccuracy in Figures 2 and 3 (the goodness‐of‐fit plots) and the corresponding Tables 3 and S3.

**FIGURE 2:**
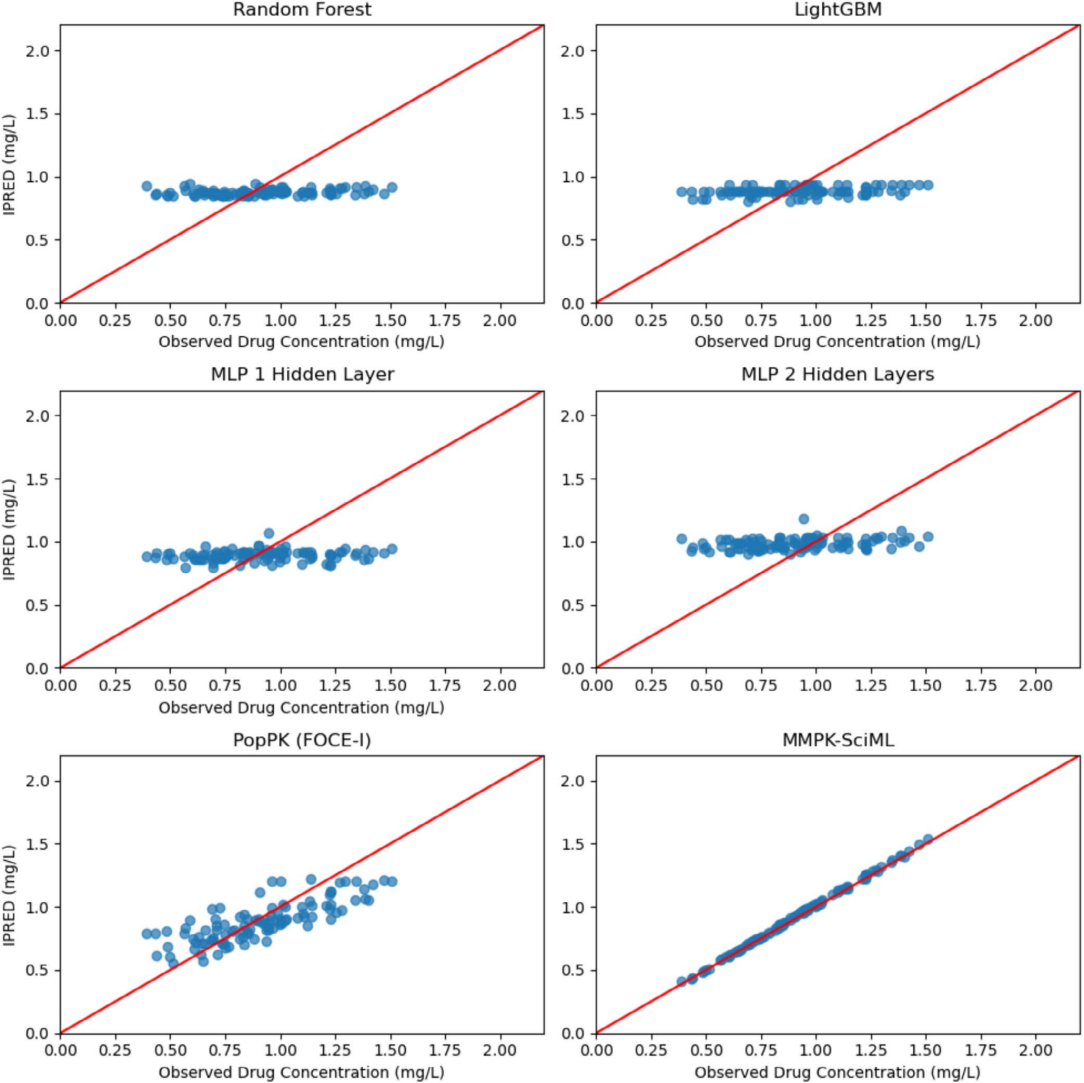
Goodness‐of‐fit (GOF) plots for the 5FU dataset showing predicted versus observed concentrations for selected trained models, with the results presented exclusively for the corresponding patients in the validation dataset.

**FIGURE 3:**
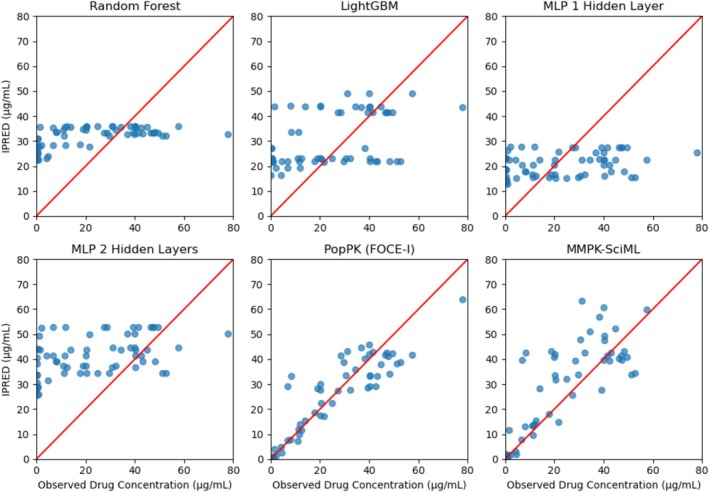
Goodness‐ of‐fit (GOF) plots for the sunitinib dataset showing predicted versus observed concentrations for selected trained models, with the results presented exclusively for the corresponding patients in the validation dataset.

**TABLE 3:** Cross validation average metrics for 5FU and sunitinib.

	5FU		Sunitinib	
Model	MAE	RMSE	MAE	RMSE
Random Forest	0.23 ± 0.02	0.32 ± 0.12	18.39 ± 2.33 (18.50 ± 2.61)	22.11 ± 3.13 (22.21 ± 3.15)
LightGBM	0.23 ± 0.03	0.32 ± 0.12	16.81 ± 1.64 (19.23 ± 2.49)	20.29 ± 1.93 (22.99 ± 3.02)
Multi‐Layer Perceptron One Hidden Layer	0.23 ± 0.02	0.32 ± 0.11	19.30 ± 2.63 (16.83 ± 2.52)	23.71 ± 4.07 (21.27 ± 2.44)
Multi‐Layer Perceptron Two Hidden Layers	0.23 ± 0.02	0.32 ± 0.11	20.82 ± 3.31 (16.59 ± 3.10)	24.96 ± 4.15 (21.49 ± 3.37)
PopPK (FOCE‐I) Simulation	0.22 ± 0.04	0.30 ± 0.12	9.69 ± 2.69	14.03 ± 3.91
PopPK (SAEM‐I) Simulation	0.21 ± 0.03	0.28 ± 0.11	9.50 ± 2.52	13.69 ± 3.65
PopPK (FOCE‐I) MAP estimation	0.14 ± 0.01	0.19 ± 0.04	**6.03 ± 1.21**	**9.07 ± 1.92**
PopPK (SAEM‐I) MAP estimation	0.14 ± 0.01	0.19 ± 0.04	6.58 ± 1.03	9.86 ± 1.45
MMPK‐SciML	**0.04 ± 0.04**	**0.08 ± 0.12**	12.55 ± 3.43	18.87 ± 5.12

*Notes:* We present the average value for the metrics (MAE: Mean Average Error, RMSE: Root Mean Square Error) and (±) the standard deviation across the 10‐cross validation. In bold we show the best performance and in brackets the results with data augmentation. Metrics are reported when using sampling from the patient‐specific distribution for test subjects.

**TABLE S3:** Full cross validation metrics for 5FU and sunitinib.

5FU—MAE												
Model	Fold 1	Fold 2	Fold 3	Fold 4	Fold 5	Fold 6	Fold 7	Fold 8	Fold 9	Fold 10	Mean	Std
Support Vector Machine	0.54	0.46	0.48	0.34	0.57	0.40	0.48	0.47	0.71	0.49	0.49	0.10
Random Forest	0.22	0.23	0.21	0.27	0.21	0.21	0.22	0.21	0.24	0.27	0.23	0.02
XGBoost	0.22	0.23	0.21	0.27	0.19	0.20	0.22	0.21	0.24	0.27	0.22	0.03
LightGBM	0.22	0.23	0.21	0.28	0.21	0.20	0.22	0.21	0.23	0.26	0.23	0.03
Multi‐Layer Perceptron (1 HL)	0.22	0.22	0.21	0.28	0.20	0.20	0.21	0.21	0.24	0.26	0.23	0.03
Multi‐Layer Perceptron (2 HL)	0.22	0.23	0.22	0.27	0.20	0.20	0.22	0.21	0.21	0.27	0.23	0.02
PopPK (FOCE‐I) Simulation	0.21	0.21	0.18	0.29	0.20	0.18	0.21	0.19	0.23	0.27	0.22	0.04
PopPK (SAEM‐I) Simulation	0.21	0.22	0.18	0.29	0.20	0.18	0.21	0.19	0.23	0.27	0.21	0.03
PopPK (FOCE‐I) MAP‐estimation	0.15	0.14	0.13	0.17	0.13	0.11	0.14	0.14	0.14	0.16	0.14	0.01
PopPK (SAEM‐I) MAP‐estimation	0.15	0.14	0.13	0.17	0.13	0.11	0.14	0.13	0.14	0.15	0.14	0.01
MMPK‐SciML	0.06	0.03	0.004	0.11	0.01	0.01	0.01	0.02	0.01	0.08	0.04	0.04
**5FU—RMSE**												
**Model**	**Fold 1**	**Fold 2**	**Fold 3**	**Fold 4**	**Fold 5**	**Fold 6**	**Fold 7**	**Fold 8**	**Fold 9**	**Fold 10**	**Mean**	**Std**
Support Vector Machine	0.61	0.53	0.55	0.56	0.64	0.47	0.57	0.55	0.77	0.73	0.60	0.09
Random Forest	0.27	0.28	0.25	0.55	0.25	0.26	0.27	0.26	0.30	0.53	0.32	0.12
XGBoost	0.27	0.28	0.25	0.55	0.24	0.25	0.27	0.26	0.30	0.54	0.32	0.12
LightGBM	0.26	0.28	0.24	0.55	0.25	0.24	0.26	0.25	0.29	0.53	0.32	0.12
Multi‐Layer Perceptron (1 HL)	0.28	0.27	0.25	0.57	0.25	0.25	0.27	0.26	0.31	0.53	0.32	0.12
Multi‐Layer Perceptron (2 HL)	0.27	0.29	0.28	0.54	0.25	0.25	0.27	0.26	0.28	0.52	0.32	0.11
PopPK (FOCE‐I) Simulation	0.25	0.26	0.21	0.54	0.23	0.23	0.26	0.23	0.29	0.52	0.30	0.12
PopPK (SAEM‐I) Simulation	0.26	0.27	0.21	0.54	0.24	0.23	0.26	0.23	0.30	0.52	0.28	0.11
PopPK (FOCE‐I) MAP estimation	0.19	0.18	0.16	0.27	0.16	0.15	0.17	0.17	0.18	0.26	0.19	0.04
PopPK (SAEM‐I) MAP estimation	0.19	0.18	0.16	0.26	0.16	0.15	0.17	0.17	0.18	0.26	0.19	0.04
MMPK‐SciML	0.13	0.04	0.01	0.29	0.02	0.01	0.01	0.02	0.02	0.30	0.08	0.12

Sunitinib—MAE												
Model	Fold 1	Fold 2	Fold 3	Fold 4	Fold 5	Fold 6	Fold 7	Fold 8	Fold 9	Fold 10	Mean	Std
Support Vector Machine	24.27	21.24	18.64	17.44	20.93	21.77	17.44	19.15	16.37	23.25	20.05	2.51
Random Forest	22.44	18.84	17.72	15.74	19.20	19.22	16.53	17.00	15.20	22.00	18.39	2.33
XGBoost	22.90	20.20	18.11	16.99	19.98	20.08	16.87	18.10	15.68	22.29	19.12	2.25
LightGBM	17.89	16.88	15.67	14.40	17.42	16.67	18.29	17.26	14.01	19.61	16.81	1.64
Multi‐Layer Perceptron (1 HL)	23.26	20.58	18.97	17.51	18.66	21.60	16.90	15.34	16.93	23.29	19.30	2.63
Multi‐Layer Perceptron (2 HL)	23.94	20.20	25.48	15.40	22.52	18.76	16.78	17.91	24.32	22.92	20.82	3.31
PopPK (FOCE‐I) Simulation	14.80	10.30	12.40	7.35	9.02	8.02	5.69	8.26	9.22	11.80	9.69	2.69
PopPK (SAEM‐I) Simulation	14.80	10.70	12.20	7.37	9.04	8.08	5.67	8.29	9.39	11.50	9.50	2.52
PopPK (FOCE‐I) MAP‐estimation	7.91	6.79	6.04	4.98	6.59	5.90	3.97	4.92	5.79	7.45	6.03	1.21
PopPK (SAEM‐I) MAP‐estimation	7.66	6.92	5.93	5.97	6.27	7.07	5.26	5.65	6.32	8.70	6.58	1.03
MMPK‐SciML	17.40	10.40	12.21	12.25	9.85	13.31	8.21	9.42	13.63	18.84	12.55	3.43
**Sunitinib—RMSE**												
**Model**	**Fold 1**	**Fold 2**	**Fold 3**	**Fold 4**	**Fold 5**	**Fold 6**	**Fold 7**	**Fold 8**	**Fold 9**	**Fold 10**	**Mean**	**Std**
Support Vector Machine	28.82	25.43	22.03	20.19	25.38	25.45	21.17	22.29	19.32	28.82	23.89	3.22
Random Forest	26.95	23.37	20.81	18.44	23.54	22.97	20.26	19.51	17.88	27.32	22.11	3.13
XGBoost	27.45	24.40	21.29	19.81	24.23	23.70	20.57	20.81	18.55	27.65	22.85	2.98
LightGBM	21.72	19.16	19.08	18.01	21.33	20.15	22.12	19.95	17.38	24.04	20.29	1.93
Multi‐Layer Perceptron (1 HL)	30.27	25.39	22.86	19.83	24.08	25.89	20.87	17.58	20.16	30.13	23.71	4.07
Multi‐Layer Perceptron (2 HL)	28.14	25.09	30.63	18.03	26.52	22.38	20.37	20.50	29.32	28.59	24.96	4.15
PopPK (FOCE‐I) Simulation	20.80	14.70	17.90	9.33	14.50	11.50	8.18	12.70	13.30	17.40	14.03	3.91
PopPK (SAEM‐I) Simulation	20.50	15.00	17.70	9.36	14.60	11.60	8.19	12.80	13.50	17.60	13.69	3.65
PopPK (FOCE‐I) MAP‐estimation	12.03	10.04	9.20	7.49	10.55	8.30	6.00	7.60	8.07	11.45	9.07	1.92
PopPK (SAEM‐I) MAP‐estimation	11.81	10.16	9.13	9.19	9.41	10.07	8.26	9.07	8.63	12.89	9.86	1.45
MMPK‐SciML	26.04	16.30	18.21	17.05	15.47	19.29	13.04	13.84	20.55	28.88	18.87	5.12

Goodness‐of‐fit plots illustrate how well a model fits the data by plotting individual predictions (IPRED) (or just predictions) against the actual observations. Upon further discussion, we realized that the IPRED shown for the Multimodal Scientific Machine Learning model were derived from maximum‐a posteriori (MAP) individual parameter estimation and those shown for the population pharmacokinetic (PopPK) models were pure simulations, that did not make use of the concentrations in the test data.

Although we stated this truthfully in our Methods section, we now realize that this comparison can be misleading. We have therefore replaced the goodness‐of‐fit plots for the PopPK model with more appropriate plots representing IPRED after MAP estimation and added the corresponding metrics to our Tables 3 and S3.

The conclusions of our article are not affected by this correction.

