# Supplementary material for: Comparing Scientific Machine Learning With Population Pharmacokinetic and Classical Machine Learning Approaches for Prediction of Drug Concentrations
Source: CPT Pharmacometrics Syst Pharmacol. 2025 Feb 7;14(4):759–69. doi: 10.1002/psp4.13313 (PMC12001275; doi:10.1002/psp4.13313)
Supplement: Supplementary file 1 — Table S1. [file PSP4-14-759-s002.docx]

**Table S1:** Description of data augmentation for sunitinib, respective distributions of training data.

| Variable | Distribution / Calculation |
| --- | --- |
| WT and HGT (by sex) | Multivariate lognormal distribution |
| Sex | Binomial distribution |
| Age | Normal distribution |
| Time after dose | Normal distribution |
| BSA | Mosteller formula  $BSA= \sqrt{\frac{(WT*HGT)}{3600}}$ |
| Dose | 50 mg (2/3 of patients) and 37.5 mg (1/3 of patients) |
| Sunitinib Concentration | Simulated from PK model using FOCE-I estimates for the respective split |

Abbreviations: WT, Weight; HT, Height; BSA, Body surface area; PK, pharmacokinetic; FOCE-I, first-order conditional estimation with interaction
