# Supplementary material for: Comparing Scientific Machine Learning With Population Pharmacokinetic and Classical Machine Learning Approaches for Prediction of Drug Concentrations
Source: CPT Pharmacometrics Syst Pharmacol. 2025 Feb 7;14(4):759–69. doi: 10.1002/psp4.13313 (PMC12001275; doi:10.1002/psp4.13313)
Supplement: Supplementary file 2 — Table S2. [file PSP4-14-759-s003.docx]

**Table S2.** Model hyperparameters for 5FU and sunitinib (Classic ML)

| **5-FU Random Forest** | | | | | | | | | | | | | | | | | | | | | | |
| --- | --- | --- | --- | --- | --- | --- | --- | --- | --- | --- | --- | --- | --- | --- | --- | --- | --- | --- | --- | --- | --- | --- |
|  | **Fold 1** | | **Fold 2** | | **Fold 3** | | **Fold 4** | | **Fold 5** | | **Fold 6** | | **Fold 7** | | **Fold 8** | | **Fold 9** | | **Fold 10** | |  | |
| n_estimators | | 776 | | 384 | | 25 | | 148 | | 563 | | 585 | | 433 | | 377 | | 282 | | 154 | |  |
| max_features | | 0.1 | | NA | | NA | | 0.1 | | NA | | NA | | NA | | 0.1 | | NA | | 0.1 | |  |
| max_depth | | 1 | | 1 | | 1 | | 1 | | 1 | | 1 | | 1 | | 1 | | 1 | | 1 | |  |
| min_samples_split | | 10 | | 15 | | 7 | | 11 | | 19 | | 4 | | 13 | | 12 | | 14 | | 20 | |  |
| min_samples_leaf | | 10 | | 5 | | 9 | | 2 | | 3 | | 8 | | 5 | | 10 | | 3 | | 3 | |  |
| MAPE | | 0.278 | | 0.291 | | 0.257 | | 0.298 | | 0.285 | | 0.260 | | 0.273 | | 0.247 | | 0.348 | | 0.247 | |  |
| RMSE | | 0.269 | | 0.284 | | 0.251 | | 0.551 | | 0.255 | | 0.263 | | 0.271 | | 0.256 | | 0.296 | | 0.534 | |  |
| MAE | | 0.219 | | 0.235 | | 0.208 | | 0.273 | | 0.210 | | 0.210 | | 0.221 | | 0.206 | | 0.238 | | 0.265 | |  |
| **5-FU Support Vector Machine** | | | | | | | | | | | | | | | | | | | | | |  |
|  |  |  |  |  |  |  |  |  |  |  |  |  |  |  |  |  |  |  |  |  |  |  |
|  | | **Fold 1** | | **Fold 2** | | **Fold 3** | | **Fold 4** | | **Fold 5** | | **Fold 6** | | **Fold 7** | | **Fold 8** | | **Fold 9** | | **Fold 10** | |  |
| C | | 3.150 | | 7.166 | | 74.181 | | 42.734 | | 67.458 | | 70.099 | | 62.558 | | 14.896 | | 61.210 | | 89.172 | |  |
| epsilon | | 0.965 | | 0.977 | | 0.997 | | 0.772 | | 1.000 | | 0.999 | | 1.000 | | 0.968 | | 0.999 | | 0.011 | |  |
| gamma | | 7.915 | | 7.911 | | 9.920 | | 0.738 | | 9.531 | | 6.906 | | 5.013 | | 8.544 | | 9.982 | | 9.400 | |  |
| MAPE | | 0.775 | | 0.622 | | 0.656 | | 0.437 | | 0.824 | | 0.529 | | 0.652 | | 0.638 | | 1.089 | | 0.516 | |  |
| RMSE | | 0.608 | | 0.526 | | 0.545 | | 0.563 | | 0.643 | | 0.470 | | 0.566 | | 0.551 | | 0.766 | | 0.734 | |  |
| MAE | | 0.538 | | 0.456 | | 0.479 | | 0.335 | | 0.571 | | 0.396 | | 0.477 | | 0.475 | | 0.714 | | 0.491 | |  |
| **5-FU Gradient Boost** | | | | | | | | | | | | | | | | | | | | | |  |
|  |  |  |  |  |  |  |  |  |  |  |  |  |  |  |  |  |  |  |  |  |  |  |
|  | | **Fold 1** | | **Fold 2** | | **Fold 3** | | **Fold 4** | | **Fold 5** | | **Fold 6** | | **Fold 7** | | **Fold 8** | | **Fold 9** | | **Fold 10** | |  |
| learning_rate | | 0.018 | | 0.013 | | 0.091 | | 0.126 | | 0.998 | | 0.997 | | 0.018 | | 0.026 | | 0.158 | | 0.145 | |  |
| min_samples_split | | 14 | | 19 | | 18 | | 14 | | 14 | | 18 | | 14 | | 15 | | 11 | | 13 | |  |
| min_samples_leaf | | 4 | | 4 | | 6 | | 5 | | 5 | | 7 | | 4 | | 4 | | 2 | | 1 | |  |
| n_estimators | | 100 | | 93 | | 54 | | 38 | | 88 | | 15 | | 100 | | 94 | | 26 | | 80 | |  |
| min_weight_fraction_leaf | | 0.499 | | 0.500 | | 0.499 | | 0.500 | | 0.023 | | 0.499 | | 0.499 | | 0.500 | | 0.006 | | 0.490 | |  |
| max_depth | | 7 | | 7 | | 6 | | 7 | | 9 | | 3 | | 7 | | 7 | | 7 | | 6 | |  |
| max_leaf_nodes | | 24 | | 20 | | 32 | | 38 | | 5 | | 49 | | 24 | | 27 | | 4 | | 21 | |  |
| max_features | | 0.130 | | 0.143 | | 0.674 | | 0.494 | | 0.706 | | 0.946 | | 0.130 | | 0.119 | | 0.157 | | 0.190 | |  |
| min_impurity_decrease | | 0.496 | | 0.412 | | 0.101 | | 0.230 | | 0.246 | | 0.226 | | 0.496 | | 0.474 | | 0.454 | | 0.478 | |  |
| MAPE | | 0.288 | | 0.280 | | 0.261 | | 0.293 | | 0.298 | | 0.242 | | 0.271 | | 0.252 | | 0.356 | | 0.248 | |  |
| RMSE | | 0.273 | | 0.283 | | 0.254 | | 0.555 | | 0.262 | | 0.246 | | 0.270 | | 0.261 | | 0.298 | | 0.538 | |  |
| MAE | | 0.225 | | 0.230 | | 0.212 | | 0.273 | | 0.217 | | 0.199 | | 0.221 | | 0.212 | | 0.241 | | 0.267 | |  |
| **5-FU Xtreme Gradient Boost** | | | | | | | | | | | | | | | | | | | | | |  |
|  |  |  |  |  |  |  |  |  |  |  |  |  |  |  |  |  |  |  |  |  |  |  |
|  | | **Fold 1** | | **Fold 2** | | **Fold 3** | | **Fold 4** | | **Fold 5** | | **Fold 6** | | **Fold 7** | | **Fold 8** | | **Fold 9** | | **Fold 10** | |  |
| learning_rate | | 0.011 | | 0.011 | | 0.010 | | 0.016 | | 0.160 | | 0.011 | | 0.011 | | 0.010 | | 0.088 | | 0.013 | |  |
| n_estimators | | 10 | | 12 | | 18 | | 18 | | 27 | | 13 | | 10 | | 17 | | 63 | | 11 | |  |
| max_depth | | 3 | | 6 | | 6 | | 7 | | 5 | | 8 | | 5 | | 8 | | 10 | | 3 | |  |
| min_child_weight | | 5 | | 6 | | 7 | | 4 | | 4 | | 4 | | 6 | | 10 | | 2 | | 1 | |  |
| subsample | | 0.520 | | 0.665 | | 0.500 | | 0.551 | | 0.804 | | 0.556 | | 0.983 | | 0.682 | | 0.987 | | 0.554 | |  |
| colsample_bytree | | 0.805 | | 0.599 | | 0.981 | | 0.696 | | 0.862 | | 0.645 | | 0.916 | | 0.861 | | 1.000 | | 0.525 | |  |
| gamma | | 0.076 | | 0.001 | | 0.164 | | 0.267 | | 0.026 | | 0.225 | | 0.063 | | 0.246 | | 0.199 | | 0.293 | |  |
| reg_lambda | | 0.310 | | 2.367 | | 1.161 | | 0.408 | | 1.695 | | 0.236 | | 0.407 | | 1.056 | | 0.821 | | 2.701 | |  |
| reg_alpha | | 0.168 | | 0.592 | | 0.112 | | 0.978 | | 0.178 | | 0.685 | | 0.058 | | 0.047 | | 0.074 | | 0.294 | |  |
| MAPE | | 0.284 | | 0.282 | | 0.253 | | 0.294 | | 0.261 | | 0.242 | | 0.268 | | 0.247 | | 0.349 | | 0.247 | |  |
| RMSE | | 0.270 | | 0.283 | | 0.247 | | 0.551 | | 0.238 | | 0.247 | | 0.266 | | 0.256 | | 0.299 | | 0.538 | |  |
| MAE | | 0.223 | | 0.231 | | 0.206 | | 0.271 | | 0.192 | | 0.199 | | 0.218 | | 0.207 | | 0.236 | | 0.267 | |  |
| **5-FU Light Gradient Boost** | | | | | | | | | | | | | | | | | | | | | |  |
|  |  |  |  |  |  |  |  |  |  |  |  |  |  |  |  |  |  |  |  |  |  |  |
|  | | **Fold 1** | | **Fold 2** | | **Fold 3** | | **Fold 4** | | **Fold 5** | | **Fold 6** | | **Fold 7** | | **Fold 8** | | **Fold 9** | | **Fold 10** | |  |
| learning_rate | | 0.143 | | 0.143 | | 0.157 | | 0.143 | | 0.143 | | 0.143 | | 0.143 | | 0.143 | | 0.143 | | 0.161 | |  |
| n_estimators | | 10 | | 10 | | 29 | | 10 | | 10 | | 10 | | 10 | | 10 | | 10 | | 75 | |  |
| max_depth | | 10 | | 10 | | 10 | | 10 | | 10 | | 10 | | 10 | | 10 | | 10 | | 9 | |  |
| num_leaves | | 41 | | 41 | | 29 | | 41 | | 41 | | 41 | | 41 | | 41 | | 41 | | 79 | |  |
| min_child_samples | | 190 | | 190 | | 176 | | 190 | | 190 | | 190 | | 190 | | 190 | | 190 | | 172 | |  |
| subsample | | 0.954 | | 0.954 | | 0.945 | | 0.954 | | 0.954 | | 0.954 | | 0.954 | | 0.954 | | 0.954 | | 0.634 | |  |
| colsample_bytree | | 0.629 | | 0.629 | | 0.820 | | 0.629 | | 0.629 | | 0.629 | | 0.629 | | 0.629 | | 0.629 | | 0.575 | |  |
| min_split_gain | | 0.191 | | 0.191 | | 0.065 | | 0.191 | | 0.191 | | 0.191 | | 0.191 | | 0.191 | | 0.191 | | 0.287 | |  |
| min_child_weight | | 1 | | 1 | | 3 | | 1 | | 1 | | 1 | | 1 | | 1 | | 1 | | 2 | |  |
| reg_lambda | | 1.154 | | 1.154 | | 0.891 | | 1.154 | | 1.154 | | 1.154 | | 1.154 | | 1.154 | | 1.154 | | 1.823 | |  |
| reg_alpha | | 0.281 | | 0.281 | | 0.091 | | 0.281 | | 0.281 | | 0.281 | | 0.281 | | 0.281 | | 0.281 | | 0.881 | |  |
| MAPE | | 0.275 | | 0.281 | | 0.251 | | 0.297 | | 0.287 | | 0.246 | | 0.270 | | 0.247 | | 0.346 | | 0.246 | |  |
| RMSE | | 0.265 | | 0.278 | | 0.244 | | 0.552 | | 0.252 | | 0.244 | | 0.264 | | 0.251 | | 0.290 | | 0.531 | |  |
| MAE | | 0.216 | | 0.227 | | 0.206 | | 0.275 | | 0.209 | | 0.201 | | 0.218 | | 0.205 | | 0.234 | | 0.264 | |  |
| **5-FU MLP one hidden layer** | | | | | | | | | | | | | | | | | | | | | |  |
|  |  |  |  |  |  |  |  |  |  |  |  |  |  |  |  |  |  |  |  |  |  |  |
|  | | **Fold 1** | | **Fold 2** | | **Fold 3** | | **Fold 4** | | **Fold 5** | | **Fold 6** | | **Fold 7** | | **Fold 8** | | **Fold 9** | | **Fold 10** | |  |
| first_layer_neurons | | 8 | | 10 | | 10 | | 7 | | 8 | | 9 | | 7 | | 5 | | 9 | | 5 | |  |
| learning_rate | | 0.012 | | 0.024 | | 0.067 | | 0.082 | | 0.031 | | 0.021 | | 0.017 | | 0.074 | | 0.029 | | 0.026 | |  |
| activation | | relu | | elu | | relu | | relu | | relu | | selu | | elu | | relu | | selu | | relu | |  |
| l1_reg | | 0.0002 | | 0.001 | | 0.0002 | | 0.00006 | | 0.011 | | 0.00005 | | 0.0007 | | 0.0007 | | 0.011 | | 0.021 | |  |
| drop_out | | 0.145 | | 0.104 | | 0.397 | | 0.125 | | 0.251 | | 0.268 | | 0.329 | | 0.059 | | 0.185 | | 0.083 | |  |
| MAPE | | 0.277 | | 0.270 | | 0.263 | | 0.280 | | 0.256 | | 0.230 | | 0.246 | | 0.253 | | 0.341 | | 0.244 | |  |
| RMSE | | 0.275 | | 0.270 | | 0.253 | | 0.571 | | 0.254 | | 0.249 | | 0.271 | | 0.255 | | 0.306 | | 0.529 | |  |
| MAE | | 0.222 | | 0.219 | | 0.212 | | 0.282 | | 0.203 | | 0.197 | | 0.214 | | 0.208 | | 0.241 | | 0.261 | |  |
| **5-FU MLP two hidden layers** | | | | | | | | | | | | | | | | | | | | | |  |
|  |  |  |  |  |  |  |  |  |  |  |  |  |  |  |  |  |  |  |  |  |  |  |
|  | | **Fold 1** | | **Fold 2** | | **Fold 3** | | **Fold 4** | | **Fold 5** | | **Fold 6** | | **Fold 7** | | **Fold 8** | | **Fold 9** | | **Fold 10** | |  |
| first_layer_neurons | | 10 | | 10 | | 5 | | 8 | | 8 | | 9 | | 8 | | 9 | | 7 | | 6 | |  |
| second_layer_neurons | | 3 | | 10 | | 7 | | 5 | | 5 | | 10 | | 3 | | 2 | | 4 | | 8 | |  |
| learning_rate | | 0.016 | | 0.024 | | 0.023 | | 0.050 | | 0.020 | | 0.032 | | 0.037 | | 0.042 | | 0.028 | | 0.024 | |  |
| activation | | selu | | relu | | elu | | elu | | elu | | elu | | relu | | relu | | elu | | elu | |  |
| l1_reg | | 0.00007 | | 0.007 | | 0.00008 | | 0.0005 | | 0.00007 | | 0.00002 | | 0.0005 | | 0.00002 | | 0.00004 | | 0.0007 | |  |
| drop_out | | 0.117 | | 0.331 | | 0.314 | | 0.266 | | 0.348 | | 0.321 | | 0.214 | | 0.312 | | 0.072 | | 0.399 | |  |
| MAPE | | 0.293 | | 0.273 | | 0.245 | | 0.306 | | 0.252 | | 0.268 | | 0.286 | | 0.272 | | 0.297 | | 0.265 | |  |
| RMSE | | 0.266 | | 0.290 | | 0.280 | | 0.543 | | 0.254 | | 0.248 | | 0.270 | | 0.259 | | 0.282 | | 0.524 | |  |
| MAE | | 0.221 | | 0.234 | | 0.224 | | 0.270 | | 0.201 | | 0.202 | | 0.225 | | 0.212 | | 0.213 | | 0.266 | |  |
| **Sunitinib Random Forest** | | | | | | | | | | | | | | | | | | | | | |  |
|  |  |  |  |  |  |  |  |  |  |  |  |  |  |  |  |  |  |  |  |  |  |  |
|  | | **Fold 1** | | **Fold 2** | | **Fold 3** | | **Fold 4** | | **Fold 5** | | **Fold 6** | | **Fold 7** | | **Fold 8** | | **Fold 9** | | **Fold 10** | |  |
| n_estimators | | 249 | | 247 | | 356 | | 19 | | 647 | | 249 | | 277 | | 363 | | 231 | | 239 | |  |
| max_features | | 0.1 | | 0.1 | | 0.1 | | 0.1 | | 0.1 | | 0.1 | | 0.1 | | 0.1 | | 0.1 | | 0.1 | |  |
| max_depth | | 1 | | 1 | | 1 | | 1 | | 1 | | 1 | | 1 | | 1 | | 1 | | 1 | |  |
| min_samples_split | | 12 | | 6 | | 12 | | 14 | | 15 | | 5 | | 8 | | 14 | | 6 | | 11 | |  |
| min_samples_leaf | | 4 | | 7 | | 4 | | 10 | | 9 | | 8 | | 10 | | 6 | | 5 | | 9 | |  |
| MAPE | | 22.304 | | 10.268 | | 21.208 | | 18.353 | | 21.332 | | 11.588 | | 8.245 | | 26.348 | | 13.763 | | 23.145 | |  |
| RMSE | | 26.954 | | 23.365 | | 20.811 | | 18.436 | | 23.539 | | 22.969 | | 20.263 | | 19.512 | | 17.884 | | 27.318 | |  |
| MAE | | 22.443 | | 18.843 | | 17.720 | | 15.737 | | 19.198 | | 19.220 | | 16.533 | | 17.001 | | 15.199 | | 22.003 | |  |
| **Sunitinib Support Vector Machine** | | | | | | | | | | | | | | | | | | | | | |  |
|  |  |  |  |  |  |  |  |  |  |  |  |  |  |  |  |  |  |  |  |  |  |  |
|  | | **Fold 1** | | **Fold 2** | | **Fold 3** | | **Fold 4** | | **Fold 5** | | **Fold 6** | | **Fold 7** | | **Fold 8** | | **Fold 9** | | **Fold 10** | |  |
| C | | 0.003 | | 0.018 | | 0.003 | | 0.018 | | 0.019 | | 0.003 | | 0.019 | | 0.018 | | 0.018 | | 0.018 | |  |
| epsilon | | 0.896 | | 0.954 | | 0.896 | | 0.745 | | 0.783 | | 0.860 | | 0.881 | | 0.745 | | 0.836 | | 0.836 | |  |
| gamma | | 7.918 | | 5.406 | | 7.918 | | 8.008 | | 7.938 | | 6.442 | | 7.938 | | 8.008 | | 9.337 | | 9.337 | |  |
| MAPE | | 28.276 | | 11.563 | | 23.684 | | 21.250 | | 26.980 | | 13.515 | | 9.907 | | 32.139 | | 14.351 | | 26.767 | |  |
| RMSE | | 28.822 | | 25.432 | | 22.028 | | 20.191 | | 25.376 | | 25.448 | | 21.168 | | 22.288 | | 19.324 | | 28.819 | |  |
| MAE | | 24.268 | | 21.235 | | 18.640 | | 17.437 | | 20.933 | | 21.774 | | 17.436 | | 19.149 | | 16.372 | | 23.254 | |  |
| **Sunitinib Gradient Boost** | | | | | | | | | | | | | | | | | | | | | |  |
|  |  |  |  |  |  |  |  |  |  |  |  |  |  |  |  |  |  |  |  |  |  |  |
|  | | **Fold 1** | | **Fold 2** | | **Fold 3** | | **Fold 4** | | **Fold 5** | | **Fold 6** | | **Fold 7** | | **Fold 8** | | **Fold 9** | | **Fold 10** | |  |
| learning_rate | | 0.010 | | 0.017 | | 0.997 | | 0.063 | | 0.017 | | 0.010 | | 0.011 | | 0.101 | | 0.174 | | 0.011 | |  |
| min_samples_split | | 14 | | 15 | | 2 | | 15 | | 15 | | 15 | | 19 | | 15 | | 16 | | 16 | |  |
| min_samples_leaf | | 2 | | 4 | | 4 | | 7 | | 4 | | 4 | | 9 | | 3 | | 3 | | 9 | |  |
| n_estimators | | 34 | | 98 | | 92 | | 90 | | 98 | | 96 | | 10 | | 72 | | 82 | | 18 | |  |
| min_weight_fraction_leaf | | 0.449 | | 0.495 | | 0.499 | | 0.499 | | 0.495 | | 0.487 | | 0.457 | | 0.499 | | 0.499 | | 0.500 | |  |
| max_depth | | 9 | | 7 | | 4 | | 5 | | 7 | | 8 | | 6 | | 6 | | 9 | | 2 | |  |
| max_leaf_nodes | | 87 | | 30 | | 16 | | 44 | | 30 | | 30 | | 21 | | 8 | | 32 | | 27 | |  |
| max_features | | 0.132 | | 0.110 | | 0.708 | | 0.157 | | 0.110 | | 0.102 | | 0.239 | | 0.220 | | 0.264 | | 0.206 | |  |
| min_impurity_decrease | | 0.177 | | 0.499 | | 0.028 | | 0.446 | | 0.499 | | 0.469 | | 0.085 | | 0.251 | | 0.355 | | 0.054 | |  |
| MAPE | | 26.915 | | 12.342 | | 24.790 | | 22.627 | | 23.751 | | 14.816 | | 9.985 | | 31.798 | | 9.598 | | 26.506 | |  |
| RMSE | | 28.634 | | 25.287 | | 22.528 | | 20.552 | | 23.446 | | 24.379 | | 21.182 | | 22.074 | | 15.937 | | 28.696 | |  |
| MAE | | 23.884 | | 20.993 | | 19.077 | | 17.569 | | 19.262 | | 20.648 | | 17.470 | | 19.013 | | 12.594 | | 23.157 | |  |
| **Sunitinib Xtreme Gradient Boost** | | | | | | | | | | | | | | | | | | | | | |  |
|  |  |  |  |  |  |  |  |  |  |  |  |  |  |  |  |  |  |  |  |  |  |  |
|  | | **Fold 1** | | **Fold 2** | | **Fold 3** | | **Fold 4** | | **Fold 5** | | **Fold 6** | | **Fold 7** | | **Fold 8** | | **Fold 9** | | **Fold 10** | |  |
| learning_rate | | 0.011 | | 0.010 | | 0.010 | | 0.010 | | 0.010 | | 0.010 | | 0.010 | | 0.010 | | 0.010 | | 0.010 | |  |
| n_estimators | | 13 | | 10 | | 10 | | 11 | | 10 | | 13 | | 14 | | 12 | | 10 | | 10 | |  |
| max_depth | | 3 | | 9 | | 8 | | 8 | | 8 | | 9 | | 9 | | 7 | | 3 | | 5 | |  |
| min_child_weight | | 8 | | 2 | | 8 | | 7 | | 10 | | 1 | | 1 | | 9 | | 7 | | 7 | |  |
| subsample | | 0.648 | | 0.634 | | 0.831 | | 0.550 | | 0.680 | | 0.721 | | 0.675 | | 0.801 | | 0.826 | | 0.819 | |  |
| colsample_bytree | | 0.711 | | 0.760 | | 0.715 | | 0.794 | | 0.983 | | 0.970 | | 0.837 | | 0.824 | | 0.914 | | 0.978 | |  |
| gamma | | 0.057 | | 0.287 | | 0.300 | | 0.273 | | 0.181 | | 0.070 | | 0.193 | | 0.257 | | 0.179 | | 0.221 | |  |
| reg_lambda | | 2.237 | | 2.577 | | 2.281 | | 2.830 | | 0.223 | | 0.270 | | 1.512 | | 2.973 | | 2.803 | | 2.123 | |  |
| reg_alpha | | 0.148 | | 0.658 | | 0.208 | | 0.002 | | 0.039 | | 0.078 | | 0.233 | | 0.905 | | 0.012 | | 0.069 | |  |
| MAPE | | 24.718 | | 11.417 | | 22.346 | | 20.877 | | 24.119 | | 13.121 | | 9.101 | | 29.041 | | 14.034 | | 24.772 | |  |
| RMSE | | 27.452 | | 24.403 | | 21.285 | | 19.805 | | 24.230 | | 23.703 | | 20.567 | | 20.808 | | 18.554 | | 27.647 | |  |
| MAE | | 22.902 | | 20.195 | | 18.110 | | 16.987 | | 19.983 | | 20.080 | | 16.872 | | 18.100 | | 15.680 | | 22.294 | |  |
| **Sunitinib Light Gradient Boost** | | | | | | | | | | | | | | | | | | | | | |  |
|  |  |  |  |  |  |  |  |  |  |  |  |  |  |  |  |  |  |  |  |  |  |  |
|  | | **Fold 1** | | **Fold 2** | | **Fold 3** | | **Fold 4** | | **Fold 5** | | **Fold 6** | | **Fold 7** | | **Fold 8** | | **Fold 9** | | **Fold 10** | |  |
| learning_rate | | 0.059 | | 0.059 | | 0.059 | | 0.059 | | 0.059 | | 0.059 | | 0.059 | | 0.059 | | 0.059 | | 0.059 | |  |
| n_estimators | | 70 | | 70 | | 70 | | 70 | | 70 | | 70 | | 70 | | 70 | | 70 | | 70 | |  |
| max_depth | | 5 | | 5 | | 5 | | 5 | | 5 | | 5 | | 5 | | 5 | | 5 | | 5 | |  |
| num_leaves | | 57 | | 57 | | 57 | | 57 | | 57 | | 57 | | 57 | | 57 | | 57 | | 57 | |  |
| min_child_samples | | 110 | | 110 | | 110 | | 110 | | 110 | | 110 | | 110 | | 110 | | 110 | | 110 | |  |
| subsample | | 0.592 | | 0.592 | | 0.592 | | 0.592 | | 0.592 | | 0.592 | | 0.592 | | 0.592 | | 0.592 | | 0.592 | |  |
| colsample_bytree | | 0.985 | | 0.985 | | 0.985 | | 0.985 | | 0.985 | | 0.985 | | 0.985 | | 0.985 | | 0.985 | | 0.985 | |  |
| min_split_gain | | 0.233 | | 0.233 | | 0.233 | | 0.233 | | 0.233 | | 0.233 | | 0.233 | | 0.233 | | 0.233 | | 0.233 | |  |
| min_child_weight | | 10 | | 10 | | 10 | | 10 | | 10 | | 10 | | 10 | | 10 | | 10 | | 10 | |  |
| reg_lambda | | 2.684 | | 2.684 | | 2.684 | | 2.684 | | 2.684 | | 2.684 | | 2.684 | | 2.684 | | 2.684 | | 2.684 | |  |
| reg_alpha | | 0.598 | | 0.598 | | 0.598 | | 0.598 | | 0.598 | | 0.598 | | 0.598 | | 0.598 | | 0.598 | | 0.598 | |  |
| MAPE | | 16.868 | | 8.374 | | 12.708 | | 12.246 | | 18.532 | | 10.712 | | 6.726 | | 20.696 | | 10.671 | | 17.693 | |  |
| RMSE | | 21.721 | | 19.164 | | 19.080 | | 18.012 | | 21.327 | | 20.150 | | 22.115 | | 19.951 | | 17.381 | | 24.044 | |  |
| MAE | | 17.890 | | 16.876 | | 15.674 | | 14.396 | | 17.423 | | 16.667 | | 18.294 | | 17.256 | | 14.009 | | 19.608 | |  |
| **Sunitinib MLP one hidden layer** | | | | | | | | | | | | | | | | | | | | | |  |
|  |  |  |  |  |  |  |  |  |  |  |  |  |  |  |  |  |  |  |  |  |  |  |
|  | | **Fold 1** | | **Fold 2** | | **Fold 3** | | **Fold 4** | | **Fold 5** | | **Fold 6** | | **Fold 7** | | **Fold 8** | | **Fold 9** | | **Fold 10** | |  |
| first_layer_neurons | | 10 | | 10 | | 10 | | 10 | | 10 | | 10 | | 9 | | 9 | | 10 | | 10 | |  |
| learning_rate | | 0.097 | | 0.097 | | 0.097 | | 0.089 | | 0.098 | | 0.097 | | 0.098 | | 0.098 | | 0.098 | | 0.097 | |  |
| activation | | selu | | selu | | selu | | selu | | selu | | selu | | selu | | selu | | selu | | selu | |  |
| l1_reg | | 0.002 | | 0.004 | | 0.002 | | 0.004 | | 0.003 | | 0.002 | | 0.002 | | 0.002 | | 0.0009 | | 0.002 | |  |
| drop_out | | 0.370 | | 0.187 | | 0.370 | | 0.387 | | 0.320 | | 0.370 | | 0.392 | | 0.238 | | 0.384 | | 0.370 | |  |
| MAPE | | 18.499 | | 9.552 | | 16.923 | | 12.777 | | 14.485 | | 7.786 | | 5.144 | | 14.561 | | 10.960 | | 20.016 | |  |
| RMSE | | 30.272 | | 25.389 | | 22.860 | | 19.828 | | 24.082 | | 25.894 | | 20.872 | | 17.579 | | 20.156 | | 30.125 | |  |
| MAE | | 23.255 | | 20.580 | | 18.971 | | 17.509 | | 18.664 | | 21.598 | | 16.901 | | 15.336 | | 16.925 | | 23.285 | |  |
| **Sunitinib MLP two hidden layers** | | | | | | | | | | | | | | | | | | | | | |  |
|  |  |  |  |  |  |  |  |  |  |  |  |  |  |  |  |  |  |  |  |  |  |  |
|  | | **Fold 1** | | **Fold 2** | | **Fold 3** | | **Fold 4** | | **Fold 5** | | **Fold 6** | | **Fold 7** | | **Fold 8** | | **Fold 9** | | **Fold 10** | |  |
| first_layer_neurons | | 10 | | 8 | | 8 | | 10 | | 9 | | 10 | | 10 | | 7 | | 8 | | 6 | |  |
| second_layer_neurons | | 10 | | 10 | | 10 | | 5 | | 7 | | 7 | | 10 | | 9 | | 10 | | 9 | |  |
| learning_rate | | 0.080 | | 0.060 | | 0.081 | | 0.065 | | 0.100 | | 0.081 | | 0.064 | | 0.082 | | 0.077 | | 0.074 | |  |
| activation | | selu | | selu | | selu | | selu | | selu | | selu | | selu | | selu | | selu | | selu | |  |
| l1_reg | | 0.00002 | | 0.0005 | | 0.00002 | | 0.00007 | | 0.002 | | 0.00002 | | 0.00002 | | 0.026 | | 0.00001 | | 0.0006 | |  |
| drop_out | | 0.229 | | 0.315 | | 0.255 | | 0.256 | | 0.313 | | 0.260 | | 0.366 | | 0.154 | | 0.213 | | 0.315 | |  |
| MAPE | | 26.444 | | 13.947 | | 35.921 | | 17.723 | | 27.554 | | 12.558 | | 7.950 | | 28.076 | | 23.749 | | 24.175 | |  |
| RMSE | | 28.136 | | 25.094 | | 30.628 | | 18.026 | | 26.522 | | 22.379 | | 20.370 | | 20.500 | | 29.320 | | 28.586 | |  |
| MAE | | 23.943 | | 20.196 | | 25.479 | | 15.404 | | 22.521 | | 18.755 | | 16.782 | | 17.907 | | 24.324 | | 22.922 | |  |
| **Sunitinib Random Forest Augmentation** | | | | | | | | | | | | | | | | | | | | | |  |
|  |  |  |  |  |  |  |  |  |  |  |  |  |  |  |  |  |  |  |  |  |  |  |
|  | | **Fold 1** | | **Fold 2** | | **Fold 3** | | **Fold 4** | | **Fold 5** | | **Fold 6** | | **Fold 7** | | **Fold 8** | | **Fold 9** | | **Fold 10** | |  |
| n_estimators | | 21 | | 21 | | 240 | | 21 | | 245 | | 21 | | 21 | | 21 | | 247 | | 21 | |  |
| max_features | | 0.1 | | 0.1 | | 0.1 | | 0.1 | | 0.1 | | 0.1 | | 0.1 | | 0.1 | | 0.1 | | 0.1 | |  |
| max_depth | | 1 | | 1 | | 1 | | 1 | | 1 | | 1 | | 1 | | 1 | | 1 | | 1 | |  |
| min_samples_split | | 2 | | 16 | | 11 | | 9 | | 9 | | 2 | | 18 | | 16 | | 12 | | 16 | |  |
| min_samples_leaf | | 6 | | 3 | | 2 | | 3 | | 2 | | 2 | | 3 | | 3 | | 2 | | 3 | |  |
| MAPE | | 25.148 | | 11.020 | | 20.797 | | 18.650 | | 22.200 | | 12.296 | | 8.255 | | 27.742 | | 13.884 | | 24.556 | |  |
| RMSE | | 27.319 | | 23.291 | | 20.461 | | 18.910 | | 23.791 | | 22.716 | | 20.101 | | 20.309 | | 17.756 | | 27.415 | |  |
| MAE | | 23.383 | | 18.738 | | 17.172 | | 16.199 | | 19.544 | | 18.991 | | 16.245 | | 17.457 | | 14.780 | | 22.456 | |  |
| **Sunitinib Support Vector Machine Augmentation** | | | | | | | | | | | | | | | | | | | | | |  |
|  |  |  |  |  |  |  |  |  |  |  |  |  |  |  |  |  |  |  |  |  |  |  |
|  | | **Fold 1** | | **Fold 2** | | **Fold 3** | | **Fold 4** | | **Fold 5** | | **Fold 6** | | **Fold 7** | | **Fold 8** | | **Fold 9** | | **Fold 10** | |  |
| C | | 0.003 | | 0.003 | | 0.003 | | 0.003 | | 0.003 | | 0.003 | | 0.003 | | 0.003 | | 0.003 | | 0.003 | |  |
| epsilon | | 0.993 | | 0.993 | | 0.993 | | 0.993 | | 0.993 | | 0.993 | | 0.937 | | 0.937 | | 0.993 | | 0.993 | |  |
| gamma | | 4.732 | | 4.732 | | 4.732 | | 4.732 | | 4.732 | | 4.732 | | 5.053 | | 5.053 | | 4.732 | | 4.732 | |  |
| MAPE | | 34.843 | | 14.937 | | 29.183 | | 27.213 | | 31.790 | | 17.975 | | 11.875 | | 37.210 | | 18.238 | | 33.523 | |  |
| RMSE | | 29.488 | | 25.521 | | 24.588 | | 22.846 | | 27.253 | | 24.771 | | 23.473 | | 25.770 | | 21.780 | | 29.194 | |  |
| MAE | | 25.823 | | 20.567 | | 20.111 | | 18.520 | | 22.456 | | 20.555 | | 19.166 | | 21.356 | | 17.359 | | 24.478 | |  |
| **Sunitinib Gradient Boost Augmentation** | | | | | | | | | | | | | | | | | | | | | |  |
|  |  |  |  |  |  |  |  |  |  |  |  |  |  |  |  |  |  |  |  |  |  |  |
|  | | **Fold 1** | | **Fold 2** | | **Fold 3** | | **Fold 4** | | **Fold 5** | | **Fold 6** | | **Fold 7** | | **Fold 8** | | **Fold 9** | | **Fold 10** | |  |
| learning_rate | | 0.033 | | 0.010 | | 0.057 | | 0.041 | | 0.093 | | 0.011 | | 0.011 | | 0.093 | | 0.036 | | 0.010 | |  |
| min_samples_split | | 5 | | 3 | | 13 | | 8 | | 13 | | 16 | | 16 | | 13 | | 18 | | 20 | |  |
| min_samples_leaf | | 2 | | 2 | | 5 | | 2 | | 5 | | 6 | | 3 | | 5 | | 8 | | 1 | |  |
| n_estimators | | 13 | | 29 | | 12 | | 10 | | 10 | | 42 | | 46 | | 10 | | 62 | | 11 | |  |
| min_weight_fraction_leaf | | 0.478 | | 0.473 | | 0.492 | | 0.500 | | 0.498 | | 0.483 | | 0.500 | | 0.498 | | 0.500 | | 0.490 | |  |
| max_depth | | 4 | | 9 | | 7 | | 9 | | 8 | | 7 | | 8 | | 8 | | 8 | | 8 | |  |
| max_leaf_nodes | | 78 | | 83 | | 50 | | 64 | | 97 | | 87 | | 82 | | 97 | | 92 | | 6 | |  |
| max_features | | 0.269 | | 0.195 | | 0.233 | | 0.234 | | 0.195 | | 0.148 | | 0.186 | | 0.195 | | 0.149 | | 0.129 | |  |
| min_impurity_decrease | | 0.380 | | 0.441 | | 0.168 | | 0.397 | | 0.256 | | 0.439 | | 0.482 | | 0.256 | | 0.234 | | 0.417 | |  |
| MAPE | | 28.808 | | 12.606 | | 23.991 | | 22.913 | | 26.257 | | 14.996 | | 9.894 | | 30.763 | | 14.022 | | 28.405 | |  |
| RMSE | | 28.810 | | 25.003 | | 22.067 | | 20.532 | | 24.807 | | 24.616 | | 21.131 | | 21.517 | | 18.364 | | 28.769 | |  |
| MAE | | 24.336 | | 20.763 | | 18.568 | | 17.379 | | 20.446 | | 20.870 | | 17.412 | | 18.586 | | 15.380 | | 23.454 | |  |
| **Sunitinib Xtreme Gradient Boost Augmentation** | | | | | | | | | | | | | | | | | | | | | |  |
|  |  |  |  |  |  |  |  |  |  |  |  |  |  |  |  |  |  |  |  |  |  |  |
|  | | **Fold 1** | | **Fold 2** | | **Fold 3** | | **Fold 4** | | **Fold 5** | | **Fold 6** | | **Fold 7** | | **Fold 8** | | **Fold 9** | | **Fold 10** | |  |
| learning_rate | | 0.016 | | 0.016 | | 0.010 | | 0.010 | | 0.016 | | 0.010 | | 0.010 | | 0.010 | | 0.016 | | 0.010 | |  |
| n_estimators | | 10 | | 10 | | 12 | | 14 | | 10 | | 10 | | 12 | | 10 | | 14 | | 12 | |  |
| max_depth | | 7 | | 7 | | 8 | | 7 | | 7 | | 8 | | 8 | | 8 | | 9 | | 8 | |  |
| min_child_weight | | 8 | | 8 | | 7 | | 6 | | 8 | | 8 | | 7 | | 8 | | 2 | | 9 | |  |
| subsample | | 0.731 | | 0.731 | | 0.779 | | 0.701 | | 0.731 | | 0.867 | | 0.779 | | 0.756 | | 0.743 | | 0.732 | |  |
| colsample_bytree | | 0.747 | | 0.758 | | 0.566 | | 0.830 | | 0.758 | | 0.790 | | 0.566 | | 0.745 | | 0.662 | | 0.844 | |  |
| gamma | | 0.282 | | 0.282 | | 0.262 | | 0.281 | | 0.282 | | 0.298 | | 0.262 | | 0.251 | | 0.122 | | 0.263 | |  |
| reg_lambda | | 2.981 | | 2.780 | | 2.819 | | 1.670 | | 2.780 | | 2.476 | | 2.819 | | 2.738 | | 2.388 | | 2.048 | |  |
| reg_alpha | | 0.533 | | 0.535 | | 0.548 | | 0.110 | | 0.535 | | 0.006 | | 0.548 | | 0.083 | | 0.194 | | 0.148 | |  |
| MAPE | | 26.215 | | 11.345 | | 22.795 | | 20.701 | | 24.363 | | 13.901 | | 9.416 | | 29.638 | | 13.609 | | 26.082 | |  |
| RMSE | | 27.578 | | 23.604 | | 21.386 | | 19.522 | | 24.146 | | 23.718 | | 20.707 | | 21.082 | | 17.904 | | 27.783 | |  |
| MAE | | 23.326 | | 19.351 | | 18.059 | | 16.661 | | 19.834 | | 20.020 | | 16.976 | | 18.277 | | 15.127 | | 22.599 | |  |
| **Sunitinib Light Gradient Boost Augmentation** | | | | | | | | | | | | | | | | | | | | | |  |
|  |  |  |  |  |  |  |  |  |  |  |  |  |  |  |  |  |  |  |  |  |  |  |
|  | | **Fold 1** | | **Fold 2** | | **Fold 3** | | **Fold 4** | | **Fold 5** | | **Fold 6** | | **Fold 7** | | **Fold 8** | | **Fold 9** | | **Fold 10** | |  |
| learning_rate | | 0.015 | | 0.010 | | 0.010 | | 0.015 | | 0.015 | | 0.015 | | 0.015 | | 0.015 | | 0.010 | | 0.010 | |  |
| n_estimators | | 10 | | 10 | | 10 | | 10 | | 10 | | 10 | | 10 | | 10 | | 10 | | 10 | |  |
| max_depth | | 6 | | 7 | | 7 | | 6 | | 3 | | 6 | | 6 | | 3 | | 7 | | 7 | |  |
| num_leaves | | 16 | | 29 | | 29 | | 16 | | 20 | | 16 | | 16 | | 20 | | 29 | | 29 | |  |
| min_child_samples | | 109 | | 46 | | 46 | | 109 | | 100 | | 109 | | 109 | | 100 | | 46 | | 46 | |  |
| subsample | | 0.896 | | 0.959 | | 0.959 | | 0.896 | | 1.000 | | 0.896 | | 0.896 | | 1.000 | | 0.959 | | 0.959 | |  |
| colsample_bytree | | 0.518 | | 0.503 | | 0.503 | | 0.518 | | 0.648 | | 0.518 | | 0.518 | | 0.648 | | 0.503 | | 0.503 | |  |
| min_split_gain | | 0.085 | | 0.175 | | 0.175 | | 0.085 | | 0.031 | | 0.085 | | 0.085 | | 0.031 | | 0.175 | | 0.175 | |  |
| min_child_weight | | 9 | | 8 | | 8 | | 9 | | 9 | | 9 | | 9 | | 9 | | 8 | | 8 | |  |
| reg_lambda | | 1.338 | | 2.596 | | 2.596 | | 1.338 | | 0.820 | | 1.338 | | 1.338 | | 0.820 | | 2.596 | | 2.596 | |  |
| reg_alpha | | 0.353 | | 0.374 | | 0.374 | | 0.353 | | 0.014 | | 0.353 | | 0.353 | | 0.014 | | 0.374 | | 0.374 | |  |
| MAPE | | 26.822 | | 12.071 | | 23.620 | | 20.658 | | 24.418 | | 13.383 | | 9.000 | | 29.292 | | 15.181 | | 26.946 | |  |
| RMSE | | 27.905 | | 24.281 | | 21.759 | | 19.587 | | 24.410 | | 23.507 | | 20.314 | | 20.804 | | 19.188 | | 28.135 | |  |
| MAE | | 23.773 | | 19.980 | | 18.311 | | 16.694 | | 20.125 | | 19.818 | | 16.650 | | 17.982 | | 16.031 | | 22.937 | |  |
| **Sunitinib MLP one hidden layer Augmentation** | | | | | | | | | | | | | | | | | | | | | |  |
|  |  |  |  |  |  |  |  |  |  |  |  |  |  |  |  |  |  |  |  |  |  |  |
|  | | **Fold 1** | | **Fold 2** | | **Fold 3** | | **Fold 4** | | **Fold 5** | | **Fold 6** | | **Fold 7** | | **Fold 8** | | **Fold 9** | | **Fold 10** | |  |
| first_layer_neurons | | 10 | | 10 | | 9 | | 9 | | 9 | | 10 | | 9 | | 10 | | 9 | | 10 | |  |
| learning_rate | | 0.099 | | 0.097 | | 0.100 | | 0.100 | | 0.098 | | 0.097 | | 0.098 | | 0.099 | | 0.098 | | 0.098 | |  |
| activation | | softplus | | softplus | | softplus | | softplus | | selu | | selu | | elu | | softplus | | selu | | elu | |  |
| l1_reg | | 0.036 | | 0.0009 | | 0.004 | | 0.002 | | 0.008 | | 0.003 | | 0.012 | | 0.075 | | 0.002 | | 0.005 | |  |
| drop_out | | 0.165 | | 0.104 | | 0.185 | | 0.054 | | 0.237 | | 0.131 | | 0.103 | | 0.199 | | 0.129 | | 0.207 | |  |
| MAPE | | 12.552 | | 8.094 | | 14.061 | | 11.256 | | 5.437 | | 1.820 | | 5.554 | | 15.495 | | 13.687 | | 10.326 | |  |
| RMSE | | 25.700 | | 21.911 | | 21.503 | | 18.827 | | 20.136 | | 19.228 | | 23.540 | | 17.735 | | 19.948 | | 24.204 | |  |
| MAE | | 22.060 | | 17.619 | | 17.513 | | 15.018 | | 14.687 | | 13.815 | | 19.164 | | 14.304 | | 15.280 | | 18.858 | |  |
| **Sunitinib MLP two hidden layers Augmentation** | | | | | | | | | | | | | | | | | | | | | |  |
|  |  |  |  |  |  |  |  |  |  |  |  |  |  |  |  |  |  |  |  |  |  |  |
|  | | **Fold 1** | | **Fold 2** | | **Fold 3** | | **Fold 4** | | **Fold 5** | | **Fold 6** | | **Fold 7** | | **Fold 8** | | **Fold 9** | | **Fold 10** | |  |
| first_layer_neurons | | 9 | | 10 | | 10 | | 10 | | 7 | | 9 | | 10 | | 10 | | 9 | | 10 | |  |
| second_layer_neurons | | 9 | | 7 | | 9 | | 7 | | 8 | | 9 | | 7 | | 9 | | 8 | | 10 | |  |
| learning_rate | | 0.086 | | 0.084 | | 0.083 | | 0.075 | | 0.085 | | 0.082 | | 0.099 | | 0.100 | | 0.073 | | 0.080 | |  |
| activation | | selu | | selu | | selu | | selu | | selu | | selu | | selu | | selu | | selu | | selu | |  |
| l1_reg | | 0.00003 | | 0.00007 | | 0.009 | | 0.0007 | | 0.00003 | | 0.00009 | | 0.00004 | | 0.099 | | 0.00002 | | 0.00001 | |  |
| drop_out | | 0.188 | | 0.324 | | 0.264 | | 0.260 | | 0.199 | | 0.152 | | 0.309 | | 0.326 | | 0.313 | | 0.328 | |  |
| MAPE | | 4.292 | | 6.031 | | 12.011 | | 2.178 | | 3.249 | | 1.378 | | 2.650 | | 3.290 | | 8.607 | | 4.648 | |  |
| RMSE | | 23.104 | | 22.057 | | 26.099 | | 21.103 | | 20.077 | | 19.694 | | 26.952 | | 15.421 | | 17.534 | | 22.852 | |  |
| MAE | | 18.645 | | 17.033 | | 21.440 | | 15.523 | | 14.168 | | 14.857 | | 21.556 | | 11.510 | | 13.922 | | 17.215 | |  |
