# Supplementary material for: Comparing Scientific Machine Learning With Population Pharmacokinetic and Classical Machine Learning Approaches for Prediction of Drug Concentrations
Source: CPT Pharmacometrics Syst Pharmacol. 2025 Feb 7;14(4):759–69. doi: 10.1002/psp4.13313 (PMC12001275; doi:10.1002/psp4.13313)
Supplement: Supplementary file 3 — Table S3. [file PSP4-14-759-s006.docx]

**Table S3.** Full cross validation metrics for 5FU and sunitinib.

| **5FU - MAE** | | | | | | | | | | | | |  |
| --- | --- | --- | --- | --- | --- | --- | --- | --- | --- | --- | --- | --- | --- |
|  |  |  |  |  |  |  |  |  |  |  |  |  |  |
| **Model** | **Fold 1** | **Fold 2** | **Fold 3** | **Fold 4** | **Fold 5** | **Fold 6** | **Fold 7** | **Fold 8** | **Fold 9** | **Fold 10** | **Mean** | **Std** |  |
| Suport Vector Machine | 0.54 | 0.46 | 0.48 | 0.34 | 0.57 | 0.40 | 0.48 | 0.47 | 0.71 | 0.49 | 0.49 | 0.10 |  |
| Random Forest | 0.22 | 0.23 | 0.21 | 0.27 | 0.21 | 0.21 | 0.22 | 0.21 | 0.24 | 0.27 | 0.23 | 0.02 |  |
| XGBoost | 0.22 | 0.23 | 0.21 | 0.27 | 0.19 | 0.20 | 0.22 | 0.21 | 0.24 | 0.27 | 0.22 | 0.03 |  |
| LightGBM | 0.22 | 0.23 | 0.21 | 0.28 | 0.21 | 0.20 | 0.22 | 0.21 | 0.23 | 0.26 | 0.23 | 0.03 |  |
| Multi-Layer Perceptron (1 HL) | 0.22 | 0.22 | 0.21 | 0.28 | 0.20 | 0.20 | 0.21 | 0.21 | 0.24 | 0.26 | 0.23 | 0.03 |  |
| Multi-Layer Perceptron (2 HL) | 0.22 | 0.23 | 0.22 | 0.27 | 0.20 | 0.20 | 0.22 | 0.21 | 0.21 | 0.27 | 0.23 | 0.02 |  |
| PopPK (FOCE-I) | 0.21 | 0.21 | 0.18 | 0.29 | 0.20 | 0.18 | 0.21 | 0.19 | 0.23 | 0.27 | 0.22 | 0.04 |  |
| PopPK (SAEM-I) | 0.21 | 0.22 | 0.18 | 0.29 | 0.20 | 0.18 | 0.21 | 0.19 | 0.23 | 0.27 | 0.21 | 0.03 |  |
| MMPK-SciML | 0.06 | 0.03 | 0.004 | 0.11 | 0.01 | 0.01 | 0.01 | 0.02 | 0.01 | 0.08 | 0.04 | 0.04 |  |
| **5FU - RMSE** | | | | | | | | | | | | |  |
|  |  |  |  |  |  |  |  |  |  |  |  |  |  |
| **Model** | **Fold 1** | **Fold 2** | **Fold 3** | **Fold 4** | **Fold 5** | **Fold 6** | **Fold 7** | **Fold 8** | **Fold 9** | **Fold 10** | **Mean** | **Std** |  |
| Suport Vector Machine | 0.61 | 0.53 | 0.55 | 0.56 | 0.64 | 0.47 | 0.57 | 0.55 | 0.77 | 0.73 | 0.60 | 0.09 |  |
| Random Forest | 0.27 | 0.28 | 0.25 | 0.55 | 0.25 | 0.26 | 0.27 | 0.26 | 0.30 | 0.53 | 0.32 | 0.12 |  |
| XGBoost | 0.27 | 0.28 | 0.25 | 0.55 | 0.24 | 0.25 | 0.27 | 0.26 | 0.30 | 0.54 | 0.32 | 0.12 |  |
| LightGBM | 0.26 | 0.28 | 0.24 | 0.55 | 0.25 | 0.24 | 0.26 | 0.25 | 0.29 | 0.53 | 0.32 | 0.12 |  |
| Multi-Layer Perceptron (1 HL) | 0.28 | 0.27 | 0.25 | 0.57 | 0.25 | 0.25 | 0.27 | 0.26 | 0.31 | 0.53 | 0.32 | 0.12 |  |
| Multi-Layer Perceptron (2 HL) | 0.27 | 0.29 | 0.28 | 0.54 | 0.25 | 0.25 | 0.27 | 0.26 | 0.28 | 0.52 | 0.32 | 0.11 |  |
| PopPK (FOCE-I) | 0.25 | 0.26 | 0.21 | 0.54 | 0.23 | 0.23 | 0.26 | 0.23 | 0.29 | 0.52 | 0.30 | 0.12 |  |
| PopPK (SAEM-I) | 0.26 | 0.27 | 0.21 | 0.54 | 0.24 | 0.23 | 0.26 | 0.23 | 0.30 | 0.52 | 0.28 | 0.11 |  |
| MMPK-SciML | 0.13 | 0.04 | 0.01 | 0.29 | 0.02 | 0.01 | 0.01 | 0.02 | 0.02 | 0.30 | 0.08 | 0.12 |  |
| **SUNITINIB - MAE** | | | | | | | | | | | | |  |
|  |  |  |  |  |  |  |  |  |  |  |  |  |  |
| **Model** | **Fold 1** | **Fold 2** | **Fold 3** | **Fold 4** | **Fold 5** | **Fold 6** | **Fold 7** | **Fold 8** | **Fold 9** | **Fold 10** | **Mean** | **Std** |  |
| Suport Vector Machine | 24.27 | 21.24 | 18.64 | 17.44 | 20.93 | 21.77 | 17.44 | 19.15 | 16.37 | 23.25 | 20.05 | 2.51 |  |
| Random Forest | 22.44 | 18.84 | 17.72 | 15.74 | 19.20 | 19.22 | 16.53 | 17.00 | 15.20 | 22.00 | 18.39 | 2.33 |  |
| XGBoost | 22.90 | 20.20 | 18.11 | 16.99 | 19.98 | 20.08 | 16.87 | 18.10 | 15.68 | 22.29 | 19.12 | 2.25 |  |
| LightGBM | 17.89 | 16.88 | 15.67 | 14.40 | 17.42 | 16.67 | 18.29 | 17.26 | 14.01 | 19.61 | 16.81 | 1.64 |  |
| Multi-Layer Perceptron (1 HL) | 23.26 | 20.58 | 18.97 | 17.51 | 18.66 | 21.60 | 16.90 | 15.34 | 16.93 | 23.29 | 19.30 | 2.63 |  |
| Multi-Layer Perceptron (2 HL) | 23.94 | 20.20 | 25.48 | 15.40 | 22.52 | 18.76 | 16.78 | 17.91 | 24.32 | 22.92 | 20.82 | 3.31 |  |
| PopPK (FOCE-I) | 14.80 | 10.30 | 12.40 | 7.35 | 9.02 | 8.02 | 5.69 | 8.26 | 9.22 | 11.80 | 9.69 | 2.69 |  |
| PopPK (SAEM-I) | 14.80 | 10.70 | 12.20 | 7.37 | 9.04 | 8.08 | 5.67 | 8.29 | 9.39 | 11.50 | 9.50 | 2.52 |  |
| MMPK-SciML | 17.40 | 10.40 | 12.21 | 12.25 | 9.85 | 13.31 | 8.21 | 9.42 | 13.63 | 18.84 | 12.55 | 3.43 |  |
| **SUNITINIB - RMSE** | | | | | | | | | | | | |  |
|  |  |  |  |  |  |  |  |  |  |  |  |  |  |
| **Model** | **Fold 1** | **Fold 2** | **Fold 3** | **Fold 4** | **Fold 5** | **Fold 6** | **Fold 7** | **Fold 8** | **Fold 9** | **Fold 10** | **Mean** | **Std** |  |
| Suport Vector Machine | 28.82 | 25.43 | 22.03 | 20.19 | 25.38 | 25.45 | 21.17 | 22.29 | 19.32 | 28.82 | 23.89 | 3.22 |  |
| Random Forest | 26.95 | 23.37 | 20.81 | 18.44 | 23.54 | 22.97 | 20.26 | 19.51 | 17.88 | 27.32 | 22.11 | 3.13 |  |
| XGBoost | 27.45 | 24.40 | 21.29 | 19.81 | 24.23 | 23.70 | 20.57 | 20.81 | 18.55 | 27.65 | 22.85 | 2.98 |  |
| LightGBM | 21.72 | 19.16 | 19.08 | 18.01 | 21.33 | 20.15 | 22.12 | 19.95 | 17.38 | 24.04 | 20.29 | 1.93 |  |
| Multi-Layer Perceptron (1 HL) | 30.27 | 25.39 | 22.86 | 19.83 | 24.08 | 25.89 | 20.87 | 17.58 | 20.16 | 30.13 | 23.71 | 4.07 |  |
| Multi-Layer Perceptron (2 HL) | 28.14 | 25.09 | 30.63 | 18.03 | 26.52 | 22.38 | 20.37 | 20.50 | 29.32 | 28.59 | 24.96 | 4.15 |  |
| PopPK (FOCE-I) | 20.80 | 14.70 | 17.90 | 9.33 | 14.50 | 11.50 | 8.18 | 12.70 | 13.30 | 17.40 | 14.03 | 3.91 |  |
| PopPK (SAEM-I) | 20.50 | 15.00 | 17.70 | 9.36 | 14.60 | 11.60 | 8.19 | 12.80 | 13.50 | 17.60 | 13.69 | 3.65 |  |
| MMPK-SciML | 26.04 | 16.30 | 18.21 | 17.05 | 15.47 | 19.29 | 13.04 | 13.84 | 20.55 | 28.88 | 18.87 | 5.12 |  |
