# Supplementary material for: Comparing Scientific Machine Learning With Population Pharmacokinetic and Classical Machine Learning Approaches for Prediction of Drug Concentrations
Source: CPT Pharmacometrics Syst Pharmacol. 2025 Feb 7;14(4):759–69. doi: 10.1002/psp4.13313 (PMC12001275; doi:10.1002/psp4.13313)
Supplement: Supplementary file 4 — Table S4. [file PSP4-14-759-s007.docx]

**Table S4.** Full cross validation populational parameters for 5FU and sunitinib (Pop-PK and MMPK-SciML)

| **5FU -** $\boldsymbol{C}\mathbf{L}$ | | | | | | | | | | | | |  |
| --- | --- | --- | --- | --- | --- | --- | --- | --- | --- | --- | --- | --- | --- |
|  |  |  |  |  |  |  |  |  |  |  |  |  |  |
| **Model** | **Fold 1** | **Fold 2** | **Fold 3** | **Fold 4** | **Fold 5** | **Fold 6** | **Fold 7** | **Fold 8** | **Fold 9** | **Fold 10** | **Mean** | **Std** |  |
| PopPK (FOCE-I) | 210.79 | 216.95 | 212.83 | 218.29 | 215.06 | 217.92 | 216.52 | 219.58 | 208.65 | 223.92 | 216.05 | 4.44 |  |
| PopPK (SAEM-I) | 207.01 | 213.33 | 208.88 | 214.89 | 211.31 | 214.25 | 212.42 | 215.60 | 205.16 | 220.15 | 212.30 | 4.42 |  |
| MMPK-SciML | 204.79 | 216.37 | 210.61 | 218.55 | 205.41 | 212.30 | 212.94 | 216.16 | 206.23 | 217.67 | 212.10 | 5.20 |  |
| **Sunitinib -** $\boldsymbol{K}_{\boldsymbol{A}}$ | | | | | | | | | | | | |  |
|  |  |  |  |  |  |  |  |  |  |  |  |  |  |
| **Model** | **Fold 1** | **Fold 2** | **Fold 3** | **Fold 4** | **Fold 5** | **Fold 6** | **Fold 7** | **Fold 8** | **Fold 9** | **Fold 10** | **Mean** | **Std** |  |
| PopPK (FOCE-I) | 0.12 | 0.13 | 0.12 | 0.17 | 0.16 | 0.11 | 0.19 | 0.17 | 0.13 | 0.23 | 0.15 | 0.04 |  |
| PopPK (SAEM-I) | 0.12 | 0.12 | 0.12 | 0.14 | 0.14 | 0.11 | 0.19 | 0.14 | 0.13 | 0.19 | 0.14 | 0.03 |  |
| MMPK-SciML | 0.27 | 0.27 | 0.32 | 0.32 | 0.31 | 0.32 | 0.30 | 0.29 | 0.31 | 0.30 | 0.30 | 0.02 |  |
| **Sunitinib -** $\boldsymbol{CL}_{\boldsymbol{S}}$ | | | | | | | | | | | | |  |
|  |  |  |  |  |  |  |  |  |  |  |  |  |  |
| **Model** | **Fold 1** | **Fold 2** | **Fold 3** | **Fold 4** | **Fold 5** | **Fold 6** | **Fold 7** | **Fold 8** | **Fold 9** | **Fold 10** | **Mean** | **Std** |  |
| PopPK (FOCE-I) | 32.92 | 33.78 | 32.34 | 32.33 | 32.93 | 34.58 | 34.46 | 31.83 | 33.28 | 32.84 | 33.13 | 0.91 |  |
| PopPK (SAEM-I) | 34.05 | 33.32 | 32.80 | 32.54 | 33.39 | 34.95 | 34.75 | 32.85 | 33.59 | 34.20 | 33.64 | 0.83 |  |
| MMPK-SciML | 35.79 | 35.87 | 35.79 | 35.78 | 35.82 | 35.86 | 35.87 | 35.78 | 35.83 | 35.73 | 35.81 | 0.05 |  |
| **Sunitinib -** $\boldsymbol{Q}_{\boldsymbol{S}}$ | | | | | | | | | | | | |  |
|  |  |  |  |  |  |  |  |  |  |  |  |  |  |
| **Model** | **Fold 1** | **Fold 2** | **Fold 3** | **Fold 4** | **Fold 5** | **Fold 6** | **Fold 7** | **Fold 8** | **Fold 9** | **Fold 10** | **Mean** | **Std** |  |
| PopPK (FOCE-I) | 0.44 | 0.34 | 0.35 | 0.33 | 0.40 | 0.37 | 0.35 | 0.33 | 0.32 | 0.38 | 0.36 | 0.04 |  |
| PopPK (SAEM-I) | 0.45 | 0.33 | 0.34 | 0.30 | 0.40 | 0.37 | 0.33 | 0.31 | 0.31 | 0.37 | 0.35 | 0.05 |  |
| MMPK-SciML | 0.47 | 0.44 | 0.46 | 0.47 | 0.46 | 0.45 | 0.46 | 0.46 | 0.45 | 0.52 | 0.47 | 0.02 |  |
| **Sunitini**b - ${\boldsymbol{V}\boldsymbol{2}}_{\boldsymbol{S}}\boldsymbol{(V}\boldsymbol{2)}$ | | | | | | | | | | | | |  |
|  |  |  |  |  |  |  |  |  |  |  |  |  |  |
| **Model** | **Fold 1** | **Fold 2** | **Fold 3** | **Fold 4** | **Fold 5** | **Fold 6** | **Fold 7** | **Fold 8** | **Fold 9** | **Fold 10** | **Mean** | **Std** |  |
| PopPK (FOCE-I) | 1732.59 | 1869.90 | 1771.65 | 1896.94 | 1779.89 | 1773.07 | 1799.90 | 1823.54 | 1878.84 | 1895.48 | 1822.18 | 59.37 |  |
| PopPK (SAEM-I) | 1695.11 | 1867.44 | 1760.54 | 1915.71 | 1776.03 | 1780.60 | 1816.28 | 1838.36 | 1886.99 | 1920.42 | 1825.75 | 73.50 |  |
| MMPK-SciML | 1339.4 | 1347.9 | 1347.0 | 1345.8 | 1334.4 | 1358.4 | 1338.5 | 1341.6 | 1344.1 | 1339.3 | 1343.64 | 6.69 |  |
| **Sunitinib -** $\boldsymbol{CL}_{\boldsymbol{M}}$ | | | | | | | | | | | | |  |
|  |  |  |  |  |  |  |  |  |  |  |  |  |  |
| **Model** | **Fold 1** | **Fold 2** | **Fold 3** | **Fold 4** | **Fold 5** | **Fold 6** | **Fold 7** | **Fold 8** | **Fold 9** | **Fold 10** | **Mean** | **Std** |  |
| PopPK (FOCE-I) | 16.84 | 16.24 | 16.69 | 16.69 | 16.46 | 16.73 | 16.51 | 15.42 | 16.62 | 16.16 | 16.44 | 0.42 |  |
| PopPK (SAEM-I) | 17.74 | 16.06 | 17.12 | 16.71 | 16.83 | 16.87 | 16.57 | 16.23 | 16.78 | 16.97 | 16.79 | 0.41 |  |
| MMPK-SciML | 10.72 | 10.62 | 10.76 | 10.58 | 10.58 | 10.99 | 11.35 | 10.88 | 10.58 | 11.04 | 10.81 | 0.26 |  |
| **Sunitinib -** $\boldsymbol{Q}_{\boldsymbol{M}}$ | | | | | | | | | | | | |  |
|  |  |  |  |  |  |  |  |  |  |  |  |  |  |
| **Model** | **Fold 1** | **Fold 2** | **Fold 3** | **Fold 4** | **Fold 5** | **Fold 6** | **Fold 7** | **Fold 8** | **Fold 9** | **Fold 10** | **Mean** | **Std** |  |
| PopPK (FOCE-I) | 3.02 | 3.22 | 3.01 | 3.94 | 3.04 | 3.47 | 3.03 | 2.96 | 3.15 | 3.23 | 3.21 | 0.30 |  |
| PopPK (SAEM-I) | 2.95 | 2.80 | 2.84 | 3.36 | 2.96 | 3.09 | 2.67 | 2.15 | 2.71 | 2.55 | 2.81 | 0.33 |  |
| MMPK-SciML | 12.59 | 12.47 | 12.63 | 12.42 | 12.42 | 12.90 | 13.33 | 12.77 | 12.42 | 12.96 | 12.69 | 0.30 |  |
| **Sunitinib -** ${\boldsymbol{V}\mathbf{2}}_{\boldsymbol{M}}\boldsymbol{(V}\boldsymbol{3)}$ | | | | | | | | | | | | |  |
|  |  |  |  |  |  |  |  |  |  |  |  |  |  |
| **Model** | **Fold 1** | **Fold 2** | **Fold 3** | **Fold 4** | **Fold 5** | **Fold 6** | **Fold 7** | **Fold 8** | **Fold 9** | **Fold 10** | **Mean** | **Std** |  |
| PopPK (FOCE-I) | 702.84 | 703.25 | 668.89 | 643.25 | 739.70 | 595.54 | 685.67 | 673.40 | 704.10 | 684.78 | 680.14 | 39.32 |  |
| PopPK (SAEM-I) | 769.97 | 655.43 | 640.28 | 616.81 | 683.43 | 588.19 | 677.33 | 760.63 | 726.01 | 754.58 | 687.27 | 63.57 |  |
| MMPK-SciML | 393.76 | 389.98 | 395.29 | 388.47 | 388.47 | 404.18 | 418.12 | 399.90 | 388.47 | 406.14 | 397.28 | 9.83 |  |
| **Sunitinib -** ${\boldsymbol{V}\boldsymbol{3}}_{\boldsymbol{M}}\boldsymbol{(V}\boldsymbol{4)}$ | | | | | | | | | | | | |  |
|  |  |  |  |  |  |  |  |  |  |  |  |  |  |
| **Model** | **Fold 1** | **Fold 2** | **Fold 3** | **Fold 4** | **Fold 5** | **Fold 6** | **Fold 7** | **Fold 8** | **Fold 9** | **Fold 10** | **Mean** | **Std** |  |
| PopPK (FOCE-I) | 641.68 | 607.78 | 641.97 | 692.27 | 624.28 | 604.73 | 594.11 | 559.19 | 616.80 | 612.88 | 619.57 | 34.97 |  |
| PopPK (SAEM-I) | 653.50 | 618.60 | 702.40 | 690.87 | 694.21 | 607.97 | 601.44 | 565.65 | 607.38 | 606.80 | 634.88 | 47.15 |  |
| MMPK-SciML | 240.76 | 238.46 | 241.69 | 237.54 | 237.54 | 247.11 | 255.61 | 244.50 | 237.54 | 248.31 | 242.91 | 5.99 |  |
