# Supplementary material for: Comparing Scientific Machine Learning With Population Pharmacokinetic and Classical Machine Learning Approaches for Prediction of Drug Concentrations
Source: CPT Pharmacometrics Syst Pharmacol. 2025 Feb 7;14(4):759–69. doi: 10.1002/psp4.13313 (PMC12001275; doi:10.1002/psp4.13313)
Supplement: Supplementary file 5 — Appendix S1. [file PSP4-14-759-s005.docx]

Comparing Scientific Machine Learning with Population Pharmacokinetic and Classical Machine Learning Approaches for Prediction of Drug Concentrations

Technical Details

Authors

Diego Valderrama^1*^_,_ Olga Teplytska^2*^, Luca Marie Koltermann^2^, Elena Trunz^3^, Eduard Schmulenson^2^, Achim Fritsch^2^, Ulrich Jaehde^2#^_,_ Holger Fröhlich^1,4#^

^1^ Department of Bioinformatics, Fraunhofer Institute for Algorithms and Scientific Computing (SCAI), Sankt Augustin, Germany

^2^ Department of Clinical Pharmacy, Institute of Pharmacy, University of Bonn, Bonn, Germany

^3^ Institute of Computer Science II, Visual Computing, University of Bonn, Bonn, Germany

^4^ Bonn-Aachen International Center for Information Technology (B-IT), University of Bonn, Bonn, Germany

*shared first authorship, ^#^shared senior authorship

# 2. Methods

## 2.2. Population pharmacokinetic modeling

For all population pharmacokinetic analyses, we used the NONMEM® version 7.5.0 and the PsN version 5.2.6. R version 4.3.1. was used in R Studio version 2023.06.1. Information on all R packages can be found in the R package list on GitHub.

## 2.3. Classical Machine Learning Algorithms

Python version 3.10 was used for all classical ML algorithms. The dependencies can be found along with the code on GitHub at <https://github.com/SCAI-BIO/MMPK-SciML> (requirements.txt). Missing data for sunitinib was imputed using the MissForest function from the missingpy library for each split for sunitinib. All algorithms were trained for 1000 epochs.

In the case of data augmentation, mean concentration values were taken from 100 simulations without including residual variability (simulated IPRED) and without re-fitting the model. One concentration value was simulated for each patient, since the classical machine learning algorithms cannot infer multiple values per patient.

## 2.4. Multimodal Pharmacokinetic SciML model (MMPK-SciML)

### 2.4.1. Variational Inference

Let $y_{it}, t\in\mathcal{T}$ denote the concentration profile measured at time points $\mathcal{T}$ for patient $i$. Furthermore, $x_{i}$ are patient-specific covariates. To simplify annotation let us define $\eta:={(\eta_{k,i})}_{k\in K,i=1,\ldots,n},$, i.e. the matrix of all random effects across all patients. From a Bayesian inference perspective, we are interested in the posterior

$$p\left( \eta\mid\{x_{i},y_{i,t}\} \right)=\frac{p(\{x_{i},y_{i,t}\}\mid\eta)p(\eta)}{p(\{x_{i},y_{i,t}\})}=\frac{p(\{x_{i},y_{i,t}\}\mid\eta)p(\eta)}{\int p\left( \{x_{i},y_{i,t}\} \mid\eta\right)p\left( \eta\right)d\eta} (S1)$$

Unfortunately, solving the integral is analytically intractable. Approximations via Markov Chain Monte Carlo techniques are possible but very time-consuming. To overcome this problem, Kingma et al. [28] introduced a stochastic variational inference framework for neural networks which allows quantification of epistemic uncertainty, i.e. uncertainty due to missing data. The key idea is to approximate $p\left( \eta\mid\{x_{i},y_{i,t}\} \right)$ by a distribution $q_{\Phi}\left( \eta\mid\{x_{i},y_{i,t}\} \right)$, which is typically supposed to be Gaussian. The means $\mu_{\eta}$ and (log) variances $\log\left( \sigma_{\eta}^{2} \right)$ of this distribution are learned from the observed data via an encoder neural network $\phi_{\theta}$:

$${\left\{ \mu_{\eta},\log\sigma_{\eta}^{2} \right\}=\left\{ \mu_{\eta_{k,i}};\log\left( \sigma_{\eta_{k,i}}^{2} \right) \right\}}_{k\in K,i=1,\ldots,n}=\phi_{\theta}\left( x_{i},\{y_{it}\} \right) (S2)$$

That means $\phi_{\theta}$ learns $|K|$-dimensional vectors $\mu_{\eta_{i}}$ and $\sigma_{\eta_{i}}^{2}$, which are different for each patient. The initial value problem can then be solved by sampling from the distribution $N\left( \mu_{\eta_{i}}, \sigma_{\eta_{i}}^{2} \right)$ which has been learned for each patient $i$ while taking advantage of the re-parametrization trick [25]. With that it is possible to formulate a loss function for training $\phi_{\theta}$ by maximizing the so-called *Evidence Lower Bound* (ELBO) on the true posterior on log-scale:

$${\log p\left( \eta\mid\{x_{i},y_{i,t}\} \right)\mathcal{\geq L}}_{ELBO}\left( \{x_{i},y_{i,t}\} \right)=\underset{data likelihood}{\underbrace{\mathbb{E}\left[ \log p\left( \left\{ x_{i},y_{i,t} \right\} \mid\eta\right) \right]}}-\underset{regularization}{\underbrace{D_{KL}\left( \left. q_{\Phi}\left( \eta| \left\{ x_{i},y_{i,t} \right\} \right) \right\|p\left( \eta\right) \right)} (S3)}$$

where $D_{KL}\left( \left. q_{\Phi}\left( \eta| \left\{ x_{i},y_{i,t} \right\} \right) \right\|p(\eta) \right)$ denotes the Kullback-Leibler divergence (statistical distance measure) between the approximate posterior distribution $q_{\Phi}\left( \eta| \left\{ x_{i},y_{i,t} \right\} \right)$ and a prior distribution $p(\eta)$. Supposing $p\left( \eta\right)=N(0,\lambda^{2})$ and Gaussian noise, the negative ELBO can be re-written as a loss function $\mathcal{l}\left( \{\tilde{y}_{i,t}\},{\{y}_{i,t}\} \right)$:

$$-\mathcal{L}_{ELBO}\left( \left\{ x_{i},y_{i,t} \right\} \right)\mathcal{\propto l}\left( \{\tilde{y}_{i,t}\},{\{y}_{i,t}\} \right):=\sum_{k\in K} \frac{1}{n}\sum_{i=1}^{n} \sum_{t\in\mathcal{T}} \frac{\left( y_{i,t}-\tilde{y}_{i,t} \right)^{2}}{2\epsilon_{i,t}^{2}}-\frac{1}{2}\left( \frac{\sigma_{\eta_{k,i}}^{2}}{\lambda^{2}}+\frac{\mu_{{\eta_{k,i}}}^{2}}{\lambda^{2}}+\log\frac{\lambda^{2}}{\sigma_{\eta_{k,i}}^{2}} \right) (S4)$$

where $\epsilon_{i,t}^{2}$ is the variance of the measurement noise and $\tilde{y}_{i,t}$ the ODE solution. For the following experiments we assumed a proportional error $\epsilon_{i,t}^{2}\propto y_{i,t}$. Note that a smaller $\lambda>0$ results in a stronger regularization of $\sigma_{\eta_{k,i}}^{2}$ and $\mu_{\eta_{k,i}}^{2}$ towards their values in the prior distribution.

### 2.4.2. Model details

### 2.4.2.1. 5FU

To learn the $\eta$ values, we defined $\phi_{\theta}$ as an encoder network consisting of three fully connected layers each of them with 64 hidden neurons, instance normalization [26] and a SoftPlus [27] activation function. The concatenation of the measured concentration, dose, weight, LBM, FM, BSA, age, sex and height was used as input for the first layer. To get $\left\{ \mu_{\eta_{k,i}};\log\left( \sigma_{\eta_{k,i}}^{2} \right) \right\}_{k\in K}$the output of the last layer was used as input for two different fully connected layers with a SoftPlus [27] activation function and $K$ outputs neurons, i.e., one layer produces $\left\{ \mu_{\eta_{k,i}} \right\}_{k\in K}$ and the other $\left\{ \log\left( \sigma_{\eta_{k,i}}^{2} \right) \right\}_{k\in K}$. We defined |$K|=1$ corresponding to the IIV for $CL$. Figure 1 (up) shows an overview of our model architecture for sunitinib.

While $\left\{ {TV}_{CL},{TV}_{V} \right\}$ were initialized as 209 L/h and 46.1 L, respectively, the weights $\theta$ of the encoder were randomly initialized. ${TV}_{V}$ was fixed. The model was trained with a batch size of 100 for 10K epochs using ADAM optimizer [25] and a learning rate of 0.001. $\lambda$ was set to 0.01 and no hyperparameter optimization was run for any model.

### 2.4.2.1. Sunitinib

$\phi_{\theta}$ was defined as a multimodal encoder containing three blocks. The first block was an encoder for static covariates $\{x_{i}\}$ consisting of 2 hidden layers, each one with 16 hidden neurons, and a SoftPlus [27] activation function. The input layer processed the sex, age, weight, height and BSA. To handle the missingness, we add an imputation layer which learns to predict the missing values before going into the input layer of our static encoder.

The second block encoded the longitudinal covariates $\{y_{it}\}$. Like the static encoder, we use an imputation layer to learn the missing values. Additionally, to be able to learn an accurate model that can handle measurements with different time sampling we used the Time-LSTM [28]. In our implementation, this model used 2 LSTM [29] layers with 64 hidden states taking the drug and metabolite concentrations, dose regimen and time since last dose as input.

The output of both encoders was concatenated and used by a third block, the projection encoder. The projection encoder had 2 different subnetworks, each consisting of 2 fully connected layers with an instance normalization in between [27] producing $|K|$ outputs which define $\left\{ \mu_{\eta_{k,i}};\log\left( \sigma_{\eta_{k,i}}^{2} \right) \right\}_{k\in K}$. We defined |$K|=4$ corresponding to the IIV for ${CL}_{S}, {V2}_{S}, F_{M}, {V2}_{M}$. Figure 1 (bottom) shows an overview of our model architecture for sunitinib.

While the weights $\theta$ of the encoder were randomly initialized, $\left\{ {TV}_{k} \right\}_{k\in J}$ were initialized using the same initial values as done by Diekstra et al. [20]: ${TV}_{K_{A}}=0.34 1/h; {TV}_{{CL}_{S}}=35.7 L/h; {TV}_{Q_{S}}=0.5 L/h; {TV}_{QH}=80 L/h$(fixed) ${TV}_{{V2}_{S}}=1360 L; {TV}_{{V3}_{S}}=588 L$ (fixed) ${TV}_{{CL}_{M}}=17.1 L/h; {TV}_{Q_{M}}=20.1 L/h; {TV}_{F_{M}}=0.21$(fixed) ${TV}_{{V2}_{M}}=635 L; {TV}_{{V3}_{M}}=388 L$ .The model was trained with the complete training patients for 500 epochs using the ADAM optimizer ​[25]​ and a learning rate of 0.01 $\lambda$ was set to 1 and no hyperparameter optimization was run for any model.

### 2.4.2.3. Learning of typical values

A subset $L$ of the population parameters ${({TV}_{k})}_{k\in L}$ are learned (see the description in our manuscript). The learnable typical value parameters are implemented via the PyTorch parameter class (torch.nn.Parameter()), which represents any parameter that can be adjusted via gradient descent. That means they are treated as if they were weights of a direct connection from an additional constant input to the model to a separate virtual output node with an identity activation function. Hence, the learnable typical value parameters ${({TV}_{k})}_{k\in L}$ can be updated during each backpropagation step due to the loss function calculation (Eq. S4).

### 2.4.3. VPC sampling procedure

For the prediction-corrected Visual Predictive checks (pcVPCs), we sample from the (approximate) posterior distribution of the random effects $\{\eta_{k,i}|k\in K,i=1,\ldots,n\}$. Note that these are represented by $n$ Gaussian densities $p_{1},p_{2},...,p_{n}$ with mean parameters $\mu_{\eta_{1}},\mu_{\eta_{2}},\ldots,\mu_{\eta_{n}}$ and standard deviations $\sigma_{\eta_{1}},\sigma_{\eta_{2}},\ldots,\sigma_{\eta_{n}}$**.**. In PK modeling it is common practice to show the median, 2.5% and 97.5% quantiles of the predicted concentrations. For this purpose, we draw 100 random samples from the approximate posteriors for each patient in the validation dataset and calculate the individual predicted concentrations (IPRED) by solving the ODE system for each patient. After this process, we estimate the overall median, 2.5% and 97.5% quantiles of the IPREDs at each time point. Furthermore, we calculated the population prediction values (PRED) values, and those were used to estimate the confidence band shown in the pcVPC plots (Figure 4). For this, we adhere to standard pharmacokinetic (PK) modeling frameworks by setting all random effects to 0 while using a patient specific dose scheme. In this case, we only use the dose and the typical values $\left\{ {TV}_{k} \right\}_{k\in J}$ for each parameter, without incorporating IIV in the predictions. We refer e.g. to Bergstand et al. [30] for details about pcVPC calculations.

Additional References

25. Kingma DP, Ba JL. Adam: A Method for Stochastic Optimization; 2014, arXiv preprint. http://arxiv.org/pdf/1412.6980.pdf1412.6980v9.

26. Ulyanov D, Vedaldi A, Lempitsky V. Instance Normalization: The Missing Ingredient for Fast Stylization; 2016, arXiv preprint. http://arxiv.org/pdf/1607.08022.pdf1607.08022v3.

27. Zheng H, Liang J, Liu W, Li Y, Yang Z. Improving deep neural networks using softplus units. In: 2015 International Joint Conference on Neural Networks (IJCNN). Improving deep neural networks using softplus units; 2015; 1–4.

28. Baytas IM, Xiao C, Zhang X, Wang F, Jain AK, Zhou J. Patient Subtyping via Time-Aware LSTM Networks. In: Proceedings of the 23rd ACM SIGKDD International Conference on Knowledge Discovery and Data Mining. Patient Subtyping via Time-Aware LSTM Networks; 2017. New York, NY: ACM; 65–74.

29. Hochreiter S, Schmidhuber J. Long Short-Term Memory. *Neural Computation* 1997; 9(8): 1735–80.

30. Bergstrand M, Hooker AC, Wallin JE, Karlsson MO. Prediction-corrected visual predictive checks for diagnosing nonlinear mixed-effects models. *AAPS J* 2011; 13(2): 143–51.
