# Supplementary figures and images for: Comparing Scientific Machine Learning With Population Pharmacokinetic and Classical Machine Learning Approaches for Prediction of Drug Concentrations
Source: CPT Pharmacometrics Syst Pharmacol. 2025 Feb 7;14(4):759–69. doi: 10.1002/psp4.13313 (PMC12001275; doi:10.1002/psp4.13313)

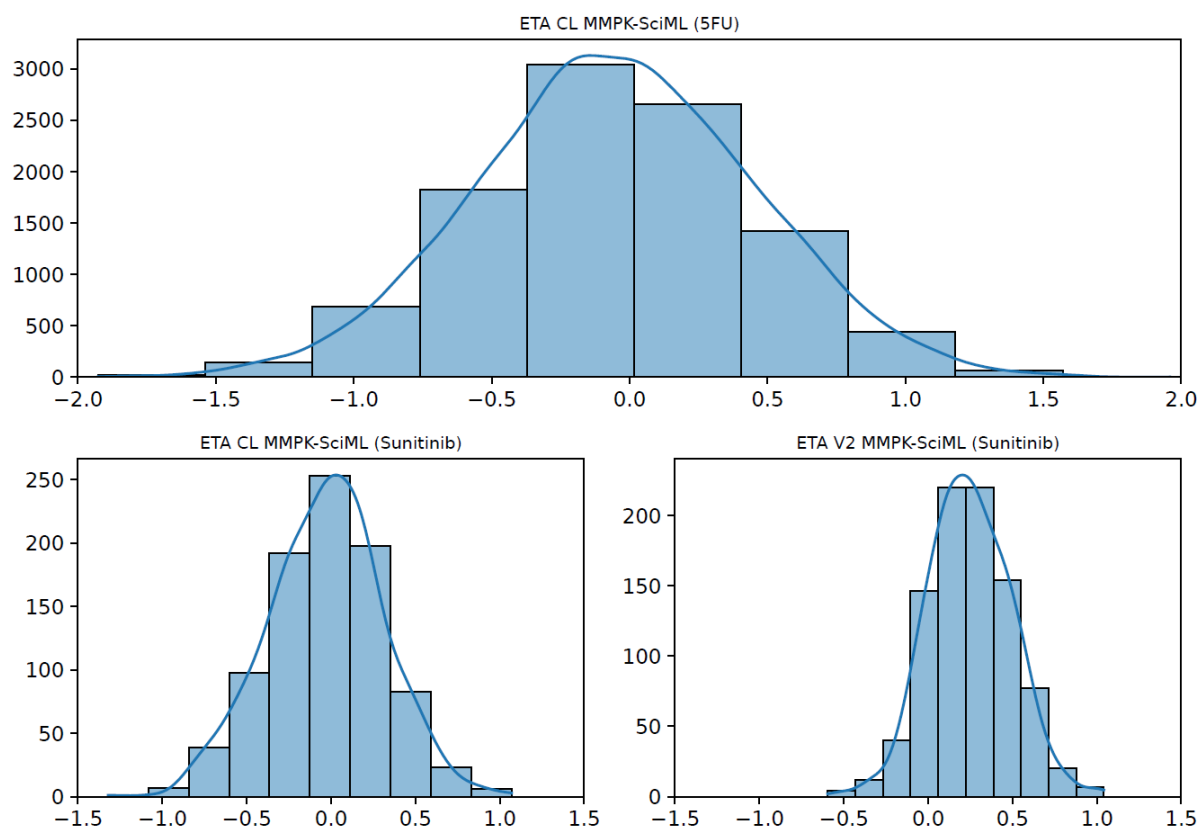

**Fig. S2.** Posterior Random effects distributions for 5FU and sunitinib datasets (MMPK-SciML)

Supplement: Supplementary file 7 — Figure S2. [file PSP4-14-759-s001.pdf]
